# Supplementary material for: Graft incompatibility between pepper and tomato elicits an immune response and triggers localized cell death
Source: Hortic Res. 2024 Sep 11;11(12):uhae255. doi: 10.1093/hr/uhae255 (PMC11630344; doi:10.1093/hr/uhae255)
Supplement: Web_Material_uhae255 [file web_material_uhae255.zip › Thomas_et_al_Hort_Res_2024_Supporting_Information_revised.docx]

## Supporting Information

The following Supporting Information is available for this article:

Fig. S1 Heterografted tomato and pepper combinations exhibit moderate survival, unstable stem integrity, and reduced growth

Fig. S2 Tomato and pepper heterografts have reduced survival and weak graft junctions.
Fig. S3 Incompatible grafts have reduced secondary growth 30 DAG.

Fig. S4 Tomato and pepper grafts were collected at 7, 14, and 21 DAG for TUNEL assays, trypan blue, and RNA-seq

Fig. S5 Non-viable tissue was quantified in ImageJ

Fig. S6 Heterografted tomato and pepper have consistent growth and persistent non-viable tissue

Fig. S7 Developmental programmed cell death is present in all graft junctions regardless of compatibility.
Fig. S8 All grafted plants have elevated programmed cell death in the graft junction

Fig. S9 All grafted plants have elevated programmed cell death in the graft junction (merged)

Fig. S10 Cross-species exudates do not affect callus growth of tomato or pepper

Fig. S11 Genetic overlap between tomato and pepper grafts shows scion-stock specificity.

Fig. S12 Scion and stock tissue have distinct upregulated genes at any given time point

Fig. S13: Incompatible graft-specific downregulated genes GO enrichment

Fig. S14: Hormonal regulation but not ROS production is upregulated in incompatible grafts

Fig. S15: RNA quality decreases over time in incompatible stocks

Fig. S16: Biological stressors upregulate distinct and shared genetic responses

Method S1 Plant material and growth conditions
Method S2 Grafting

Method S3 Pepper Compatibility Grafts

Method S4 Propidium Iodide Staining

Method S5 Bend Test

Method S6 Instron three-point bend test

Method S7 DAMP Assay

Method S8 Trypan Blue staining

Method S9 TUNEL Assay

Method S10 RNA-sequencing and bioinformatic processing

Method S11 Orthogroup Parsing

Method S12 Comparative Transcriptomics

Method S13 Statistical Analysis

Table S1 Graft survival overtime and manual bend tests
Table S2 Phenotypic data from pepper and tomato compatibility screen

Table S3 Instron 3-point bend test results and statistical analysis

Table S4 Non-viable tissue data and statistical analysis

Table S5 Wound exudate and DAMP assay on callus growth and statistical analysis

Table S6 *Solanum lycopersicum* (tomato) raw read counts

Table S7 Ca*psicum annuum* (pepper) raw read counts

Table S8 Tomato Wald Test output

Table S9 Pepper Wald Test output

Table S10 Genes with upregulation in heterografts based on likelihood ratio test

Table S11 Genes downregulated in heterografts based on likelihood ratio test

Table S12 GO term enrichment of genes upregulated in heterografted plants as determined by likelihood ratio testing

Table S13 GO term enrichment of genes downregulated in heterografted plants as determined by likelihood ratio testing

Table S14 Genes involved in processes of interest which were used to generate heatmaps

Table S15 Alignment rate of RNA-seq libraries

Table S16 Orthogrouping of TAIR10 (Arabidopsis), ITAG4 (tomato), and CM334 (pepper)

Table S17 Shared Orthogroups

Table S18 GO term enrichment from orthogroup overlap

Table S19 Genes upregulated following biological stressors

Table S20 Genetic overlap and statistical analysis

Table S21 GO enrichment of genes overlap between grafting and biological stressors

Table S22 GO enrichment for heterograft-specific upregulated genes


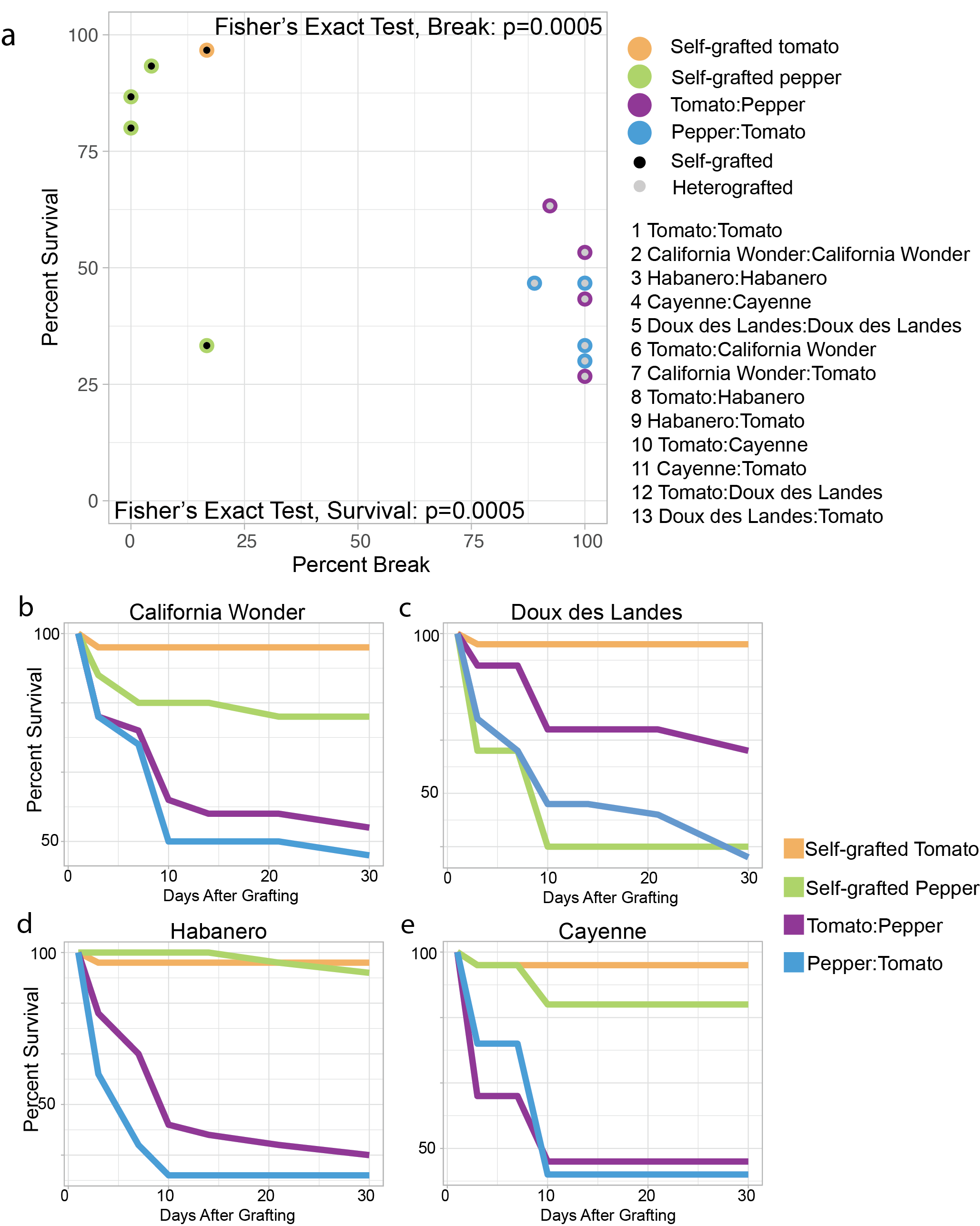


**Figure S1: Heterografted tomato and pepper combinations exhibit reduced survival and junction stability**. The relationship between percent survival (y-axis) and percent break (x-axis) is shown for all graft combinations (a). Black dots denote self-grafts, grey dots denote heterografts. Self-grafted tomato is outlined in orange. Self-grafted pepper is outlined in green. Heterografts where the scion is tomato are outlined in purple. Heterografts where the stock is tomato are outlined in blue. The identity of each data point is labeled 1-13. Percent survival n=30; For bend test sample size see Table S1. (b-e) The survival of grafted plants 0 to 30 DAG. (b) Survival of self-grafted tomato and self-and heterografted *Capsicum annuum var. California Wonder* 0-30 DAG. (c) Survival of self-grafted tomato and self-and heterografted *Capsicum annuum var. Doux des Landes* 0-30 DAG. (d) Survival for self-grafted tomato and self-and heterografted *Capsicum chinense var. Habanero* 0-30 DAG. (e) Survival of self-grafted tomato and self-and heterografted *Capsicum annuum var. Cayenne* 0-30 DAG. Self-grafted tomato survival is shown by an orange line. Self-grafted pepper survival is shown by a green line. Survival of heterografts, where the scion is tomato, is shown as a purple line. Survival of heterografts, where the stock is tomato, is shown as a blue line. n = 30 for b-e.


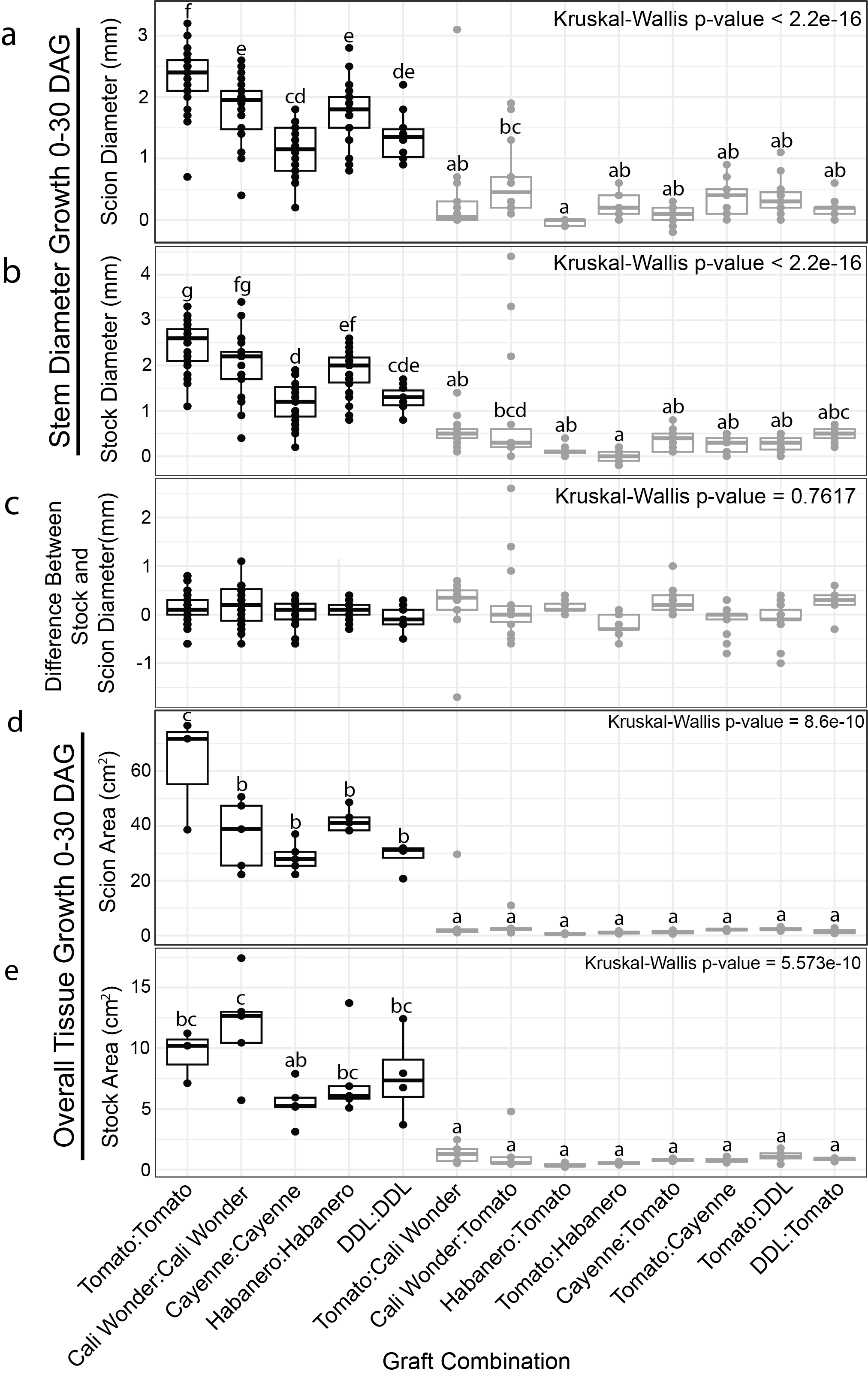


### Figure S2: Tomato and pepper heterografts have reduced growth 30 DAG.

(a) The change in stem diameter 2 cm above the graft site between 0 and 30 DAG (scion). (b) The change in stem diameter is 2 cm below the graft site between 0 and 30 DAG (stock). (c) The difference between the stock and scion diameter at 30 DAG (differential growth). (d)The 2D area of the entire shoot (including leaves) 30 DAG (scion). (e) The 2D area of the stock (including the entire root system) 30 DAG (stock). California Wonder abbreviated to Cali Wonder, Doux des Landes abbreviated to DDL. Biological replicates are depicted as jitter and described in detail in Table S2. Self-grafted plants shown in black, heterografted plants shown in grey. Kruskal–Wallis one-way analysis of variance was used to detect significant differences between self-and heterografted combinations, p-value <0.05. Compact letter display based on adjusted p-value of Tukey’s HSD Test.


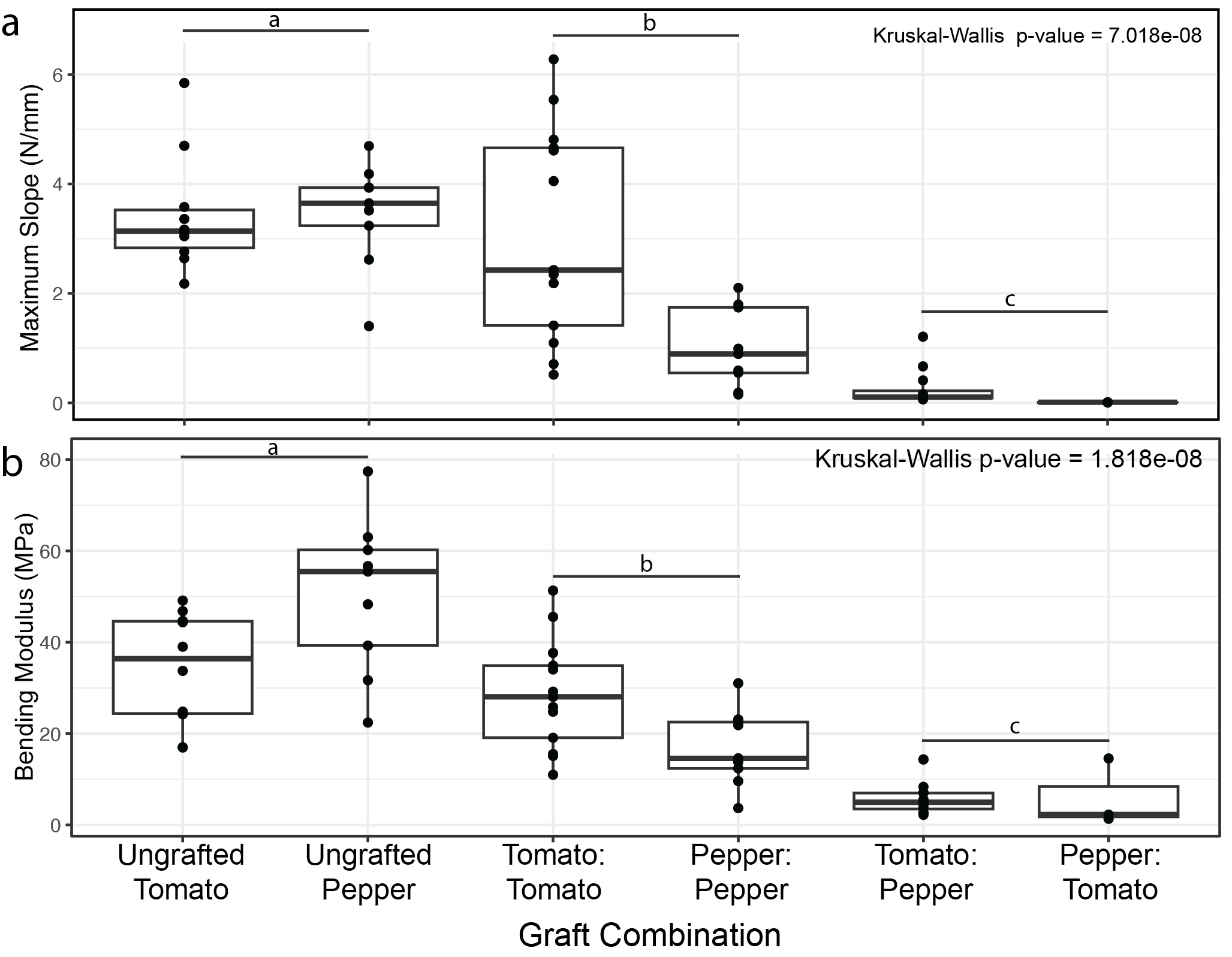


### Figure S3: Tomato and pepper heterografts have weak graft junctions.

(a) The maximum slope of the force-displacement (force/distance) curve required to bend the stem or graft junction during 3-point bend tests. (b) The Bending Modulus (MPa) required to bend the junction for each sample in the 3-Point Bend Test. Kruskal–Wallis one-way analysis of variance was used to detect significant differences between graft types (ungrafted, self-grafted, heterografted), Wilcoxon signed-rank test was used to determine pairwise comparison and compact letter display; p-value <0.05. Biological replicates are depicted as jitter as well as described in detail in Methods S6.


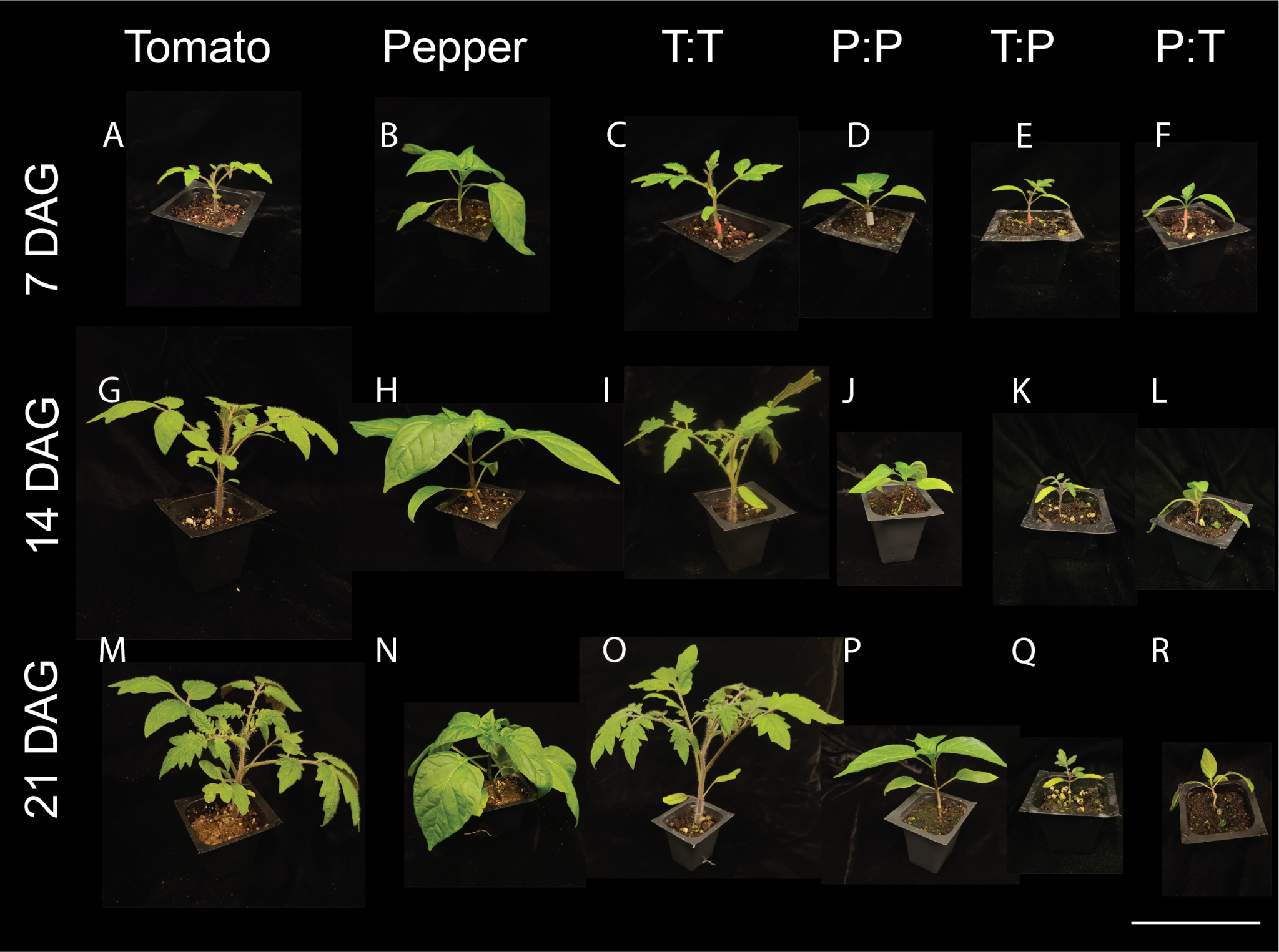


### Figure S4: Tomato and pepper grafts were collected at 7, 14, and 21 DAG for TUNEL assays, trypan blue, and RNA-seq

(a-r) Tomato (*Solanum lycopersicum var. M82*) and Pepper (*Capsicum annuum var. California Wonder*) were utilized for TUNEL assays, trypan blue staining, and RNA-seq. Ungrafted tomato, the same age as the grafted plants were collected 7 (a), 14 (g), and 21 DAG (m). Ungrafted pepper, the same age as the grafted plants were collected 7 (b), 14 (h), and 21 DAG (n). Self-grafted tomato junctions were collected 7 (c), 14 (i), and 21 DAG (o). Self-grafted pepper junctions were collected 7 (d), 14 (j), and 21 DAG (p). Tomato:pepper graft junctions were collected 7 (e), 14 (k), and 21 DAG (q). Pepper:tomato graft junctions were collected 7 (f), 14 (l), and 21 DAG (r). All plants are set equal with a scale bar = 10 cm.


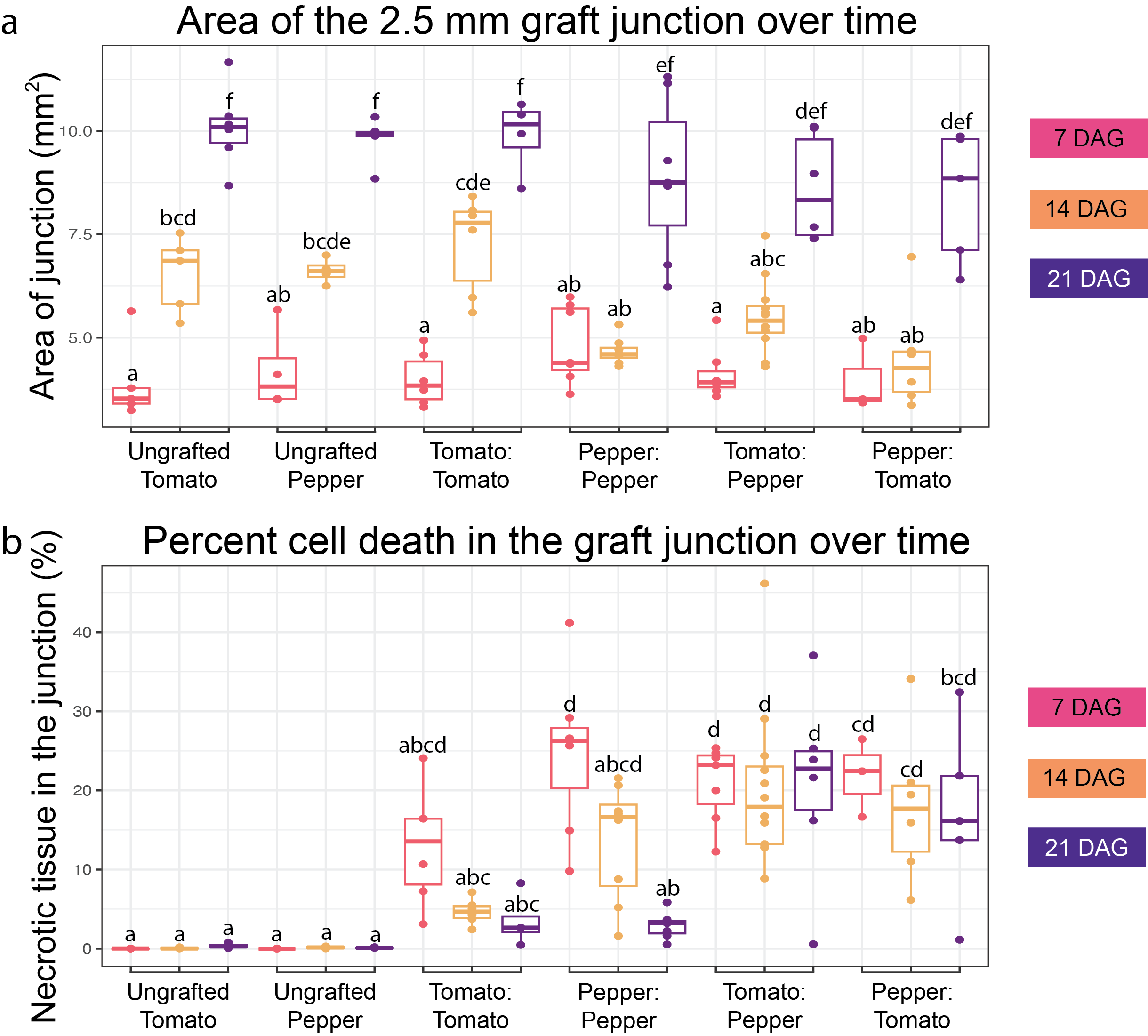


### Figure S5: Heterografted tomato and pepper have consistent growth and persistent non-viable tissue

(a) The area of the tissue analyzed and (b) the percent of non-viable tissue from the ungrafted stem, self-grafted junctions, and heterografted junctions at 7, 14, and 21 DAG. All tissue was 2.5 cm long, centered around the graft site; the samples differed in width. Pink points are 7 DAG, orange points are 14 DAG, and purple points are 21 DAG. Compact letter display based on adjusted p-value of Tukey’s HSD Test. Biological replicates are depicted as jitter as well as described in detail in Table S4


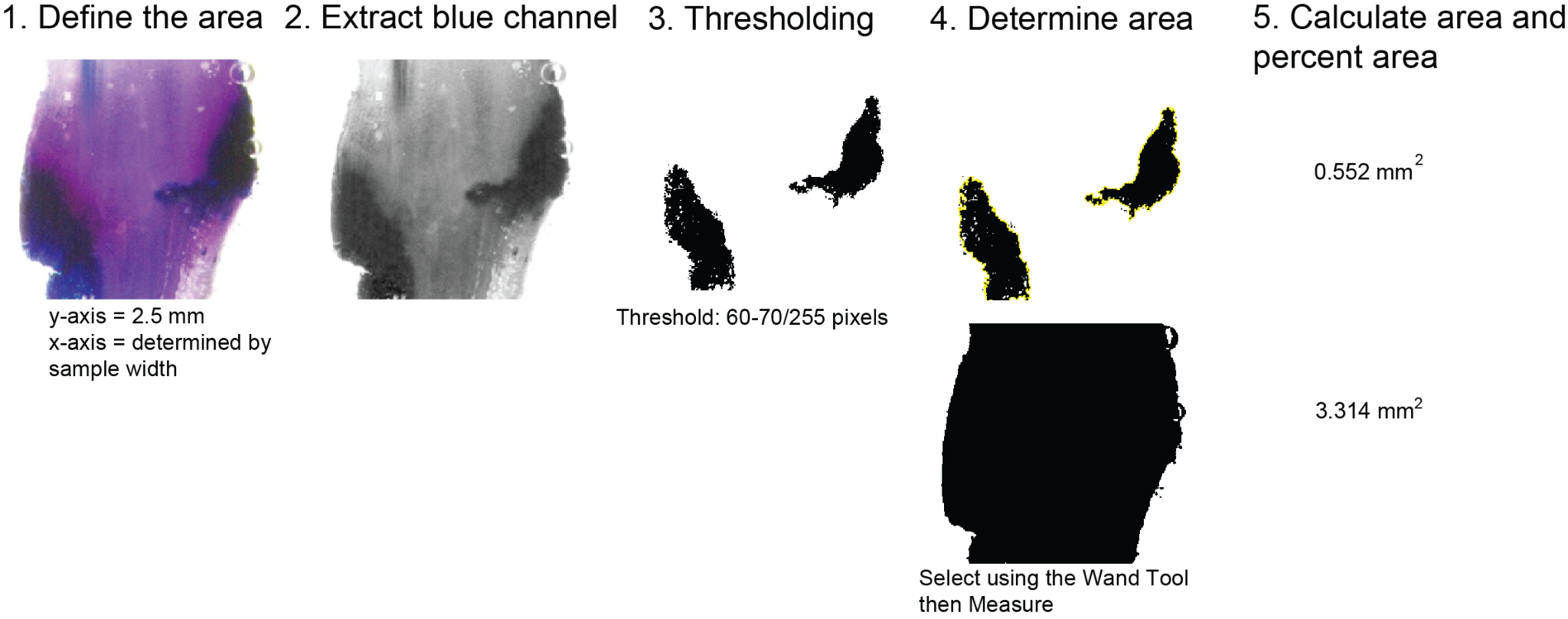


### Figure S6: Non-viable tissue was quantified in ImageJ.

Tissue stained with Trypan blue was cut transversely and imaged immediately. Images of graft junctions were used to extract 2.5 mm long images (1). The width of the tissue varied by sample. The blue channel was extracted (Image →Color→ Split Channels; 2). Using the blue channel, thresholding was applied (Image →Adjust→ Threshold; 3) with a cut-off of 60-70 out of 255 bit. The remaining segments were selected using the wand tool and measured (Analyze →Measure; 4). The resulting area can be used to calculate the area of non-viable tissue (5). By determining the total area of the tissue with the blue channel (4), the percentage of the junction consisting of non-viable tissue can also be calculated (5).


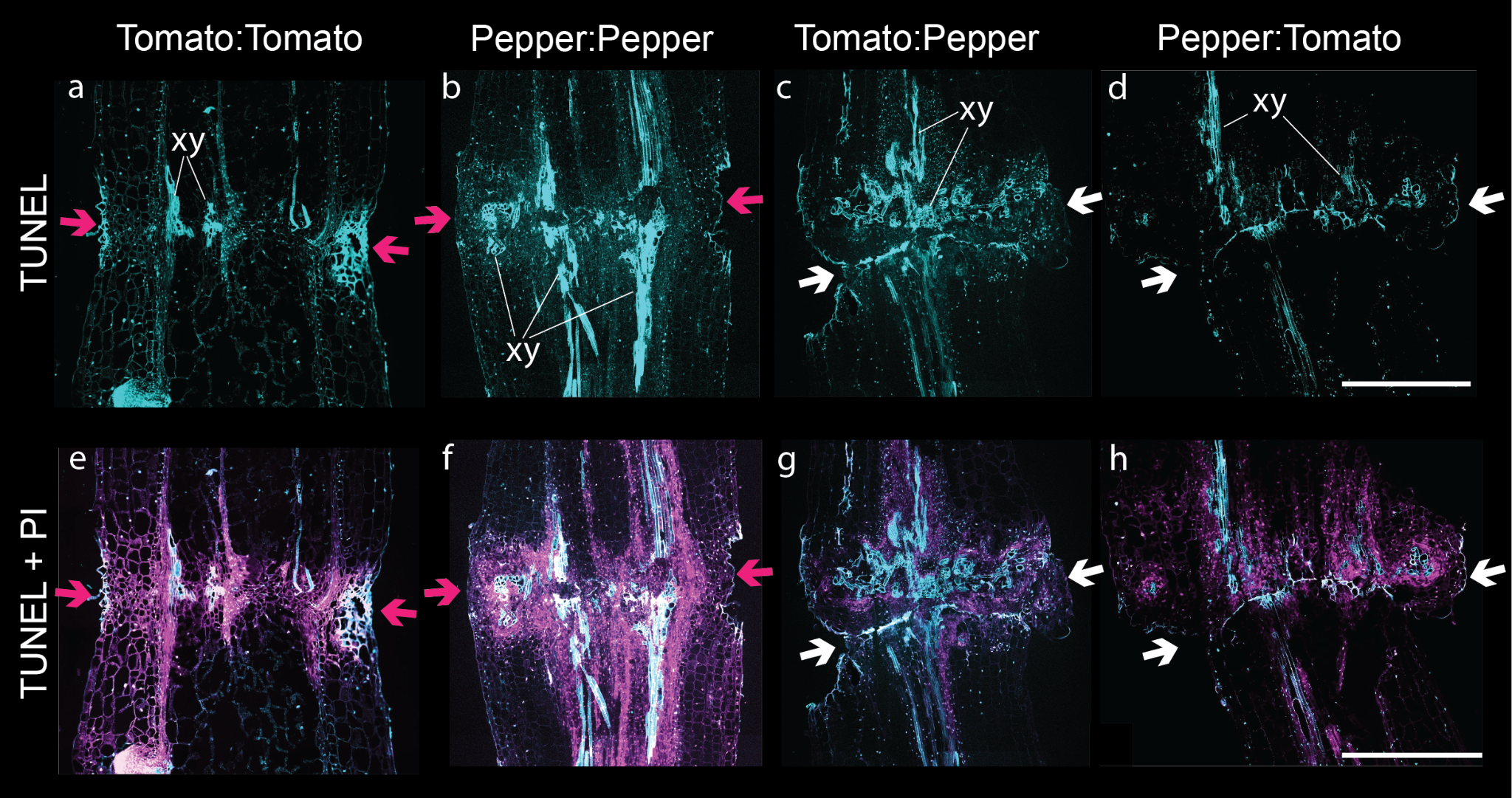


**Figure S7: Developmental programmed cell death is present in all graft junctions regardless of compatibility.**A representative graft junction from (a,e) tomato:tomato, (b,f) pepper:pepper, (c,g) tomato:pepper, (d,h) pepper:tomato 14 DAG. (a-d) TUNEL fluorescein-12-dUTP-labeled DNA and autofluorescence are false-colored cyan. (e-h) the TUNEL fluorescence merged with propidium iodide (false-colored magenta) staining nucleic acid and cell walls. Pink arrows indicate a successful graft junction with healed xylem, and white arrows indicate a failed vascular reconnection. Examples of newly developed xylem are labeled (xy). All images are equal and the scale bar is 500 µm.


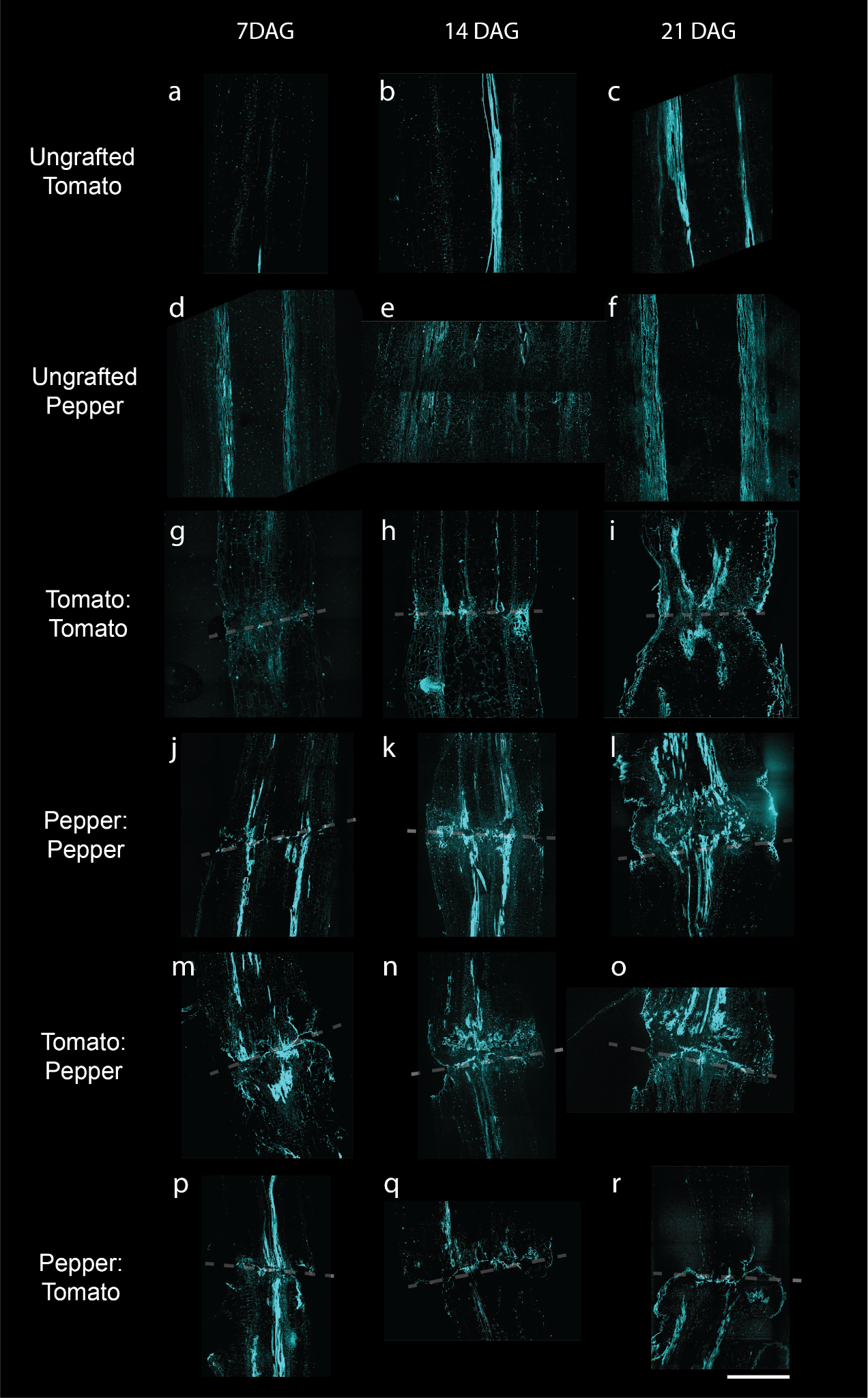


### Figure S8: All grafted plants have elevated programmed cell death in the graft junction

(a-r) Representative micrographs of TdT dUTP Nick-End Labeling (TUNEL) on different tissues and graft types. A representative micrograph of ungrafted tomato stem at 7 DAG (a), 14 DAG (b), and 21 DAG (c). Representative micrographs of ungrafted pepper stem at 7 DAG (d), 14 DAG (e), and 21 DAG (f). Representative micrographs of self-graft tomato junctions at 7 DAG (g), 14 DAG (h), and 21 DAG (i). Representative micrographs of self-graft pepper junctions at 7 DAG (j), 14 DAG (k), and 21 DAG (l). Representative micrographs of tomato:pepper junctions at 7 DAG (m), 14 DAG (n), and 21 DAG (o). Representative micrographs of pepper:tomato junctions at 7 DAG (p), 14 DAG (q), and 21 DAG (r). Fluorescence is fluorescein-12-dUTP-labeled DNA and autofluorescence from xylem; false-colored cyan. All images are equal, and the scale bar is 500 um.


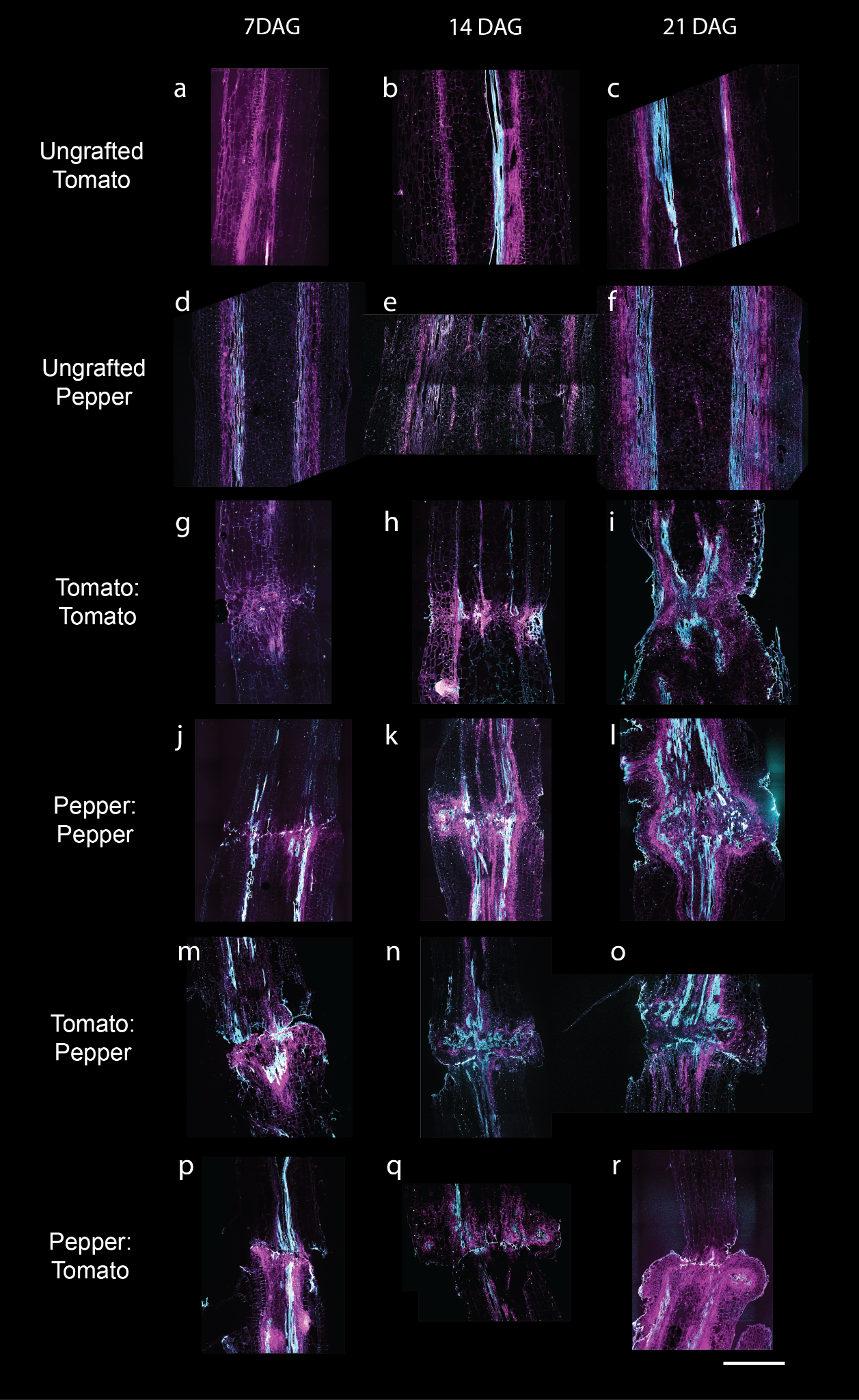


### Figure S9: All grafted plants have elevated programmed cell death in the graft junction (merged).

(a-r) Representative micrographs of TdT dUTP Nick-End Labeling (TUNEL) and propidium iodide background staining on different tissue and graft types. A representative micrograph of ungrafted tomato stem at 7 DAG (a), 14 DAG (b), and 21 DAG (c). Representative micrographs of ungrafted pepper stem at 7 DAG (d), 14 DAG (e), and 21 DAG (f). Representative micrographs of self-graft tomato junctions at 7 DAG (g), 14 DAG (h), and 21 DAG (i). Representative micrographs of self-graft pepper junctions at 7 DAG (j), 14 DAG (k), and 21 DAG (l). Representative micrographs of tomato:pepper junctions at 7 DAG (m), 14 DAG (n), and 21 DAG (o). Representative micrographs of pepper:tomato junctions at 7 DAG (p), 14 DAG (q), and 21 DAG (r). Cyan fluorescence is fluorescein-12-dUTP-labeled DNA and autofluorescence, false-colored. Magenta fluorescence is propidium iodide which stains all nucleic acids and cell walls, false-colored. All images are equal and the scale bar is = 500 um.


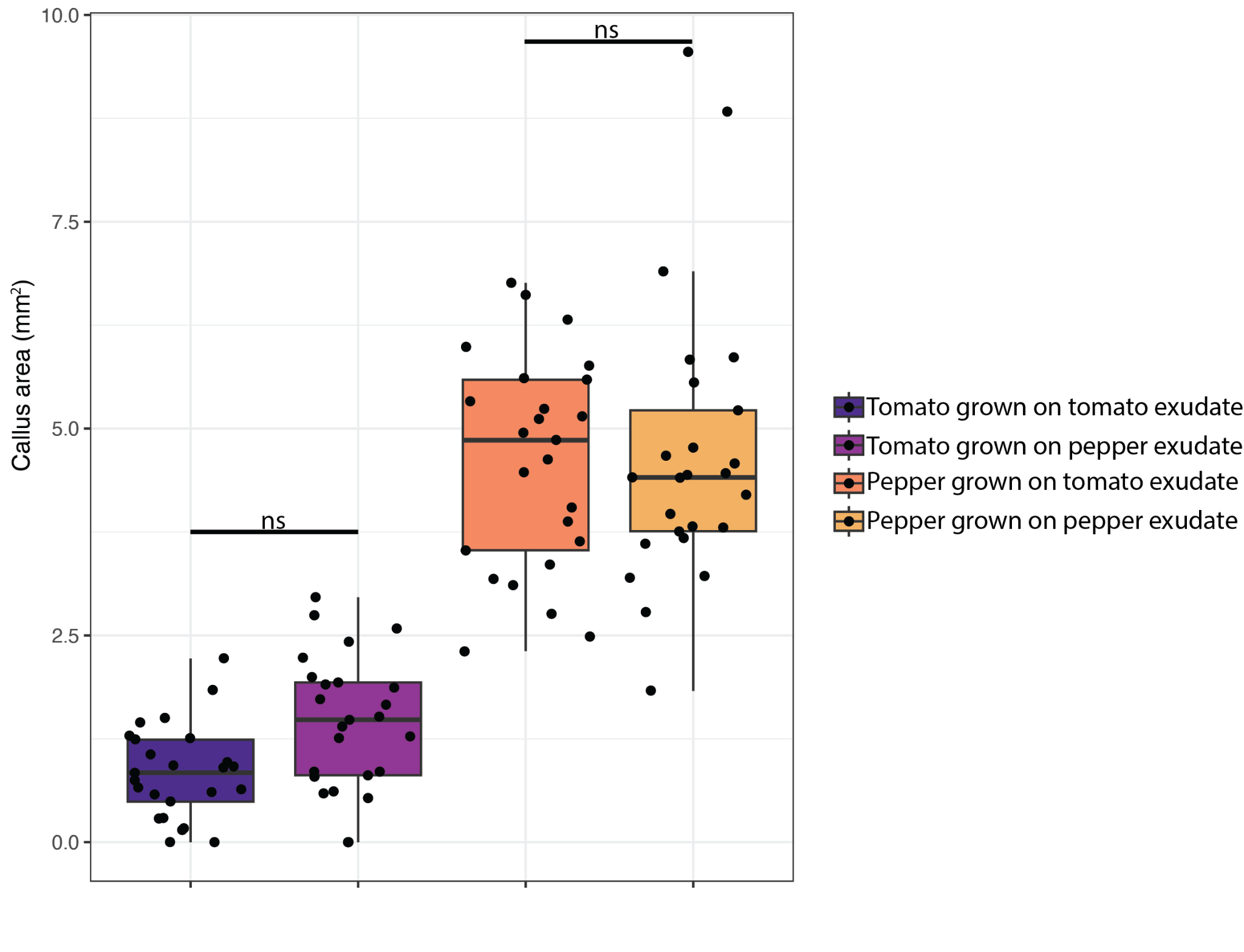


### Figure S10: Cross-species exudates do not affect callus growth of tomato or pepper

The area of tomato and pepper hypocotyl explants cultured on media containing either tomato or pepper exudates. Dark purple points are tomato callus grown on media containing tomato exudates. Magenta points are tomato callus grown on media containing pepper exudates. Orange points are pepper grown on media containing tomato exudates. Yellow points are pepper grown on media containing pepper exudates. Statistical analysis was conducted using Paired T-test, p< 0.05. n=25 for each combination. Biological replicates are depicted as jitter as well as described in detail in Table S5.


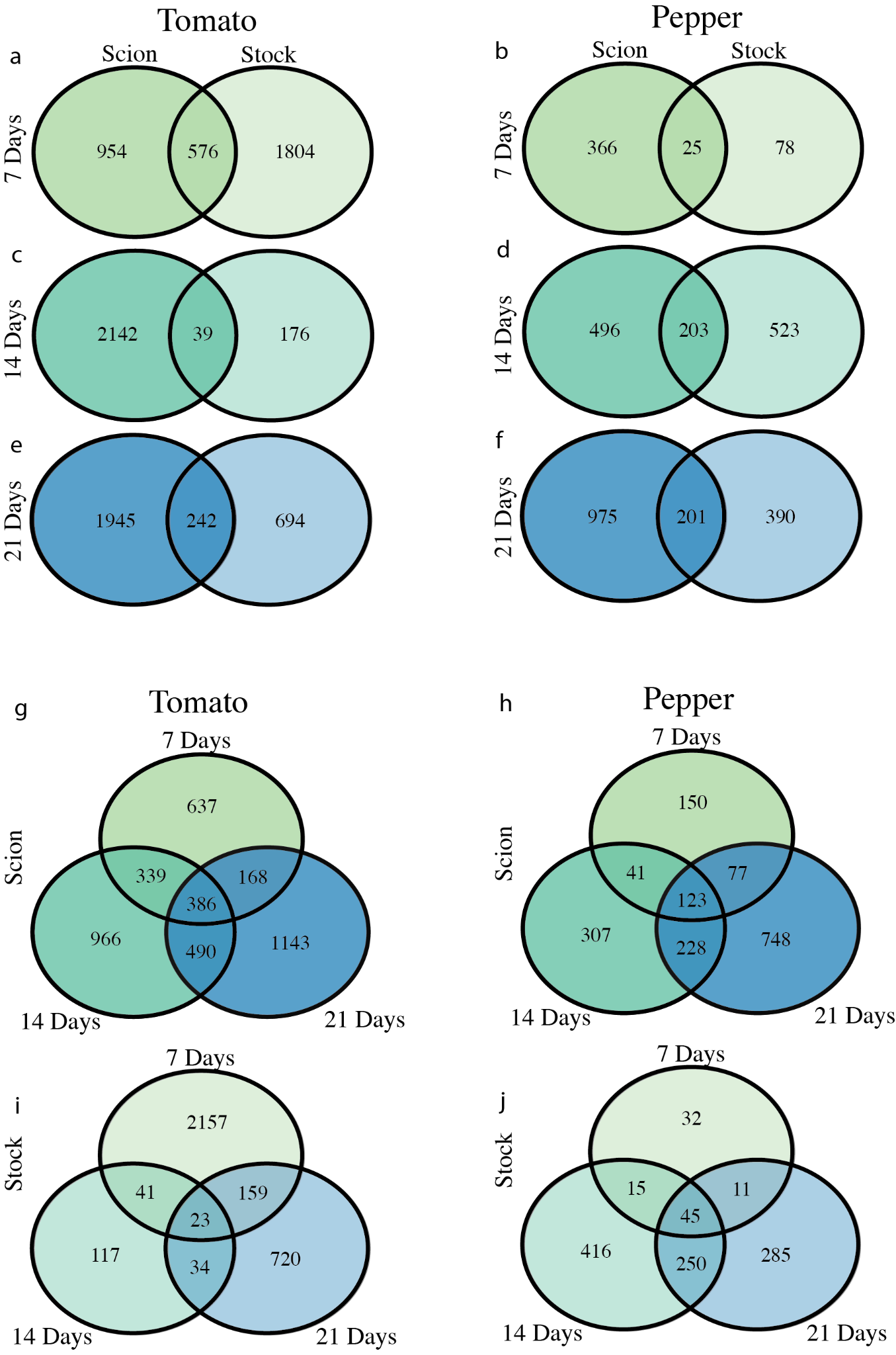


### Figure S11: Genetic overlap between tomato and pepper grafts shows scion-stock specificity.

The overlap of genes uniquely upregulated in heterografted tissue (based on likelihood ratio testing, p<0.05) (a-j). The overlap between the tomato scion-stock tissue (a) and pepper scion-stock at 7 DAG (b). The overlap between the tomato scion-stock tissue (c) and pepper scion-stock at 14 DAG (d). The overlap between the tomato scion-stock tissue (e) and pepper scion-stock at 21 DAG (f). The overlap between 7, 14, and 21 DAG was determined for tomato scion (g), pepper scion (h) tomato stock (i), and pepper stock (j).


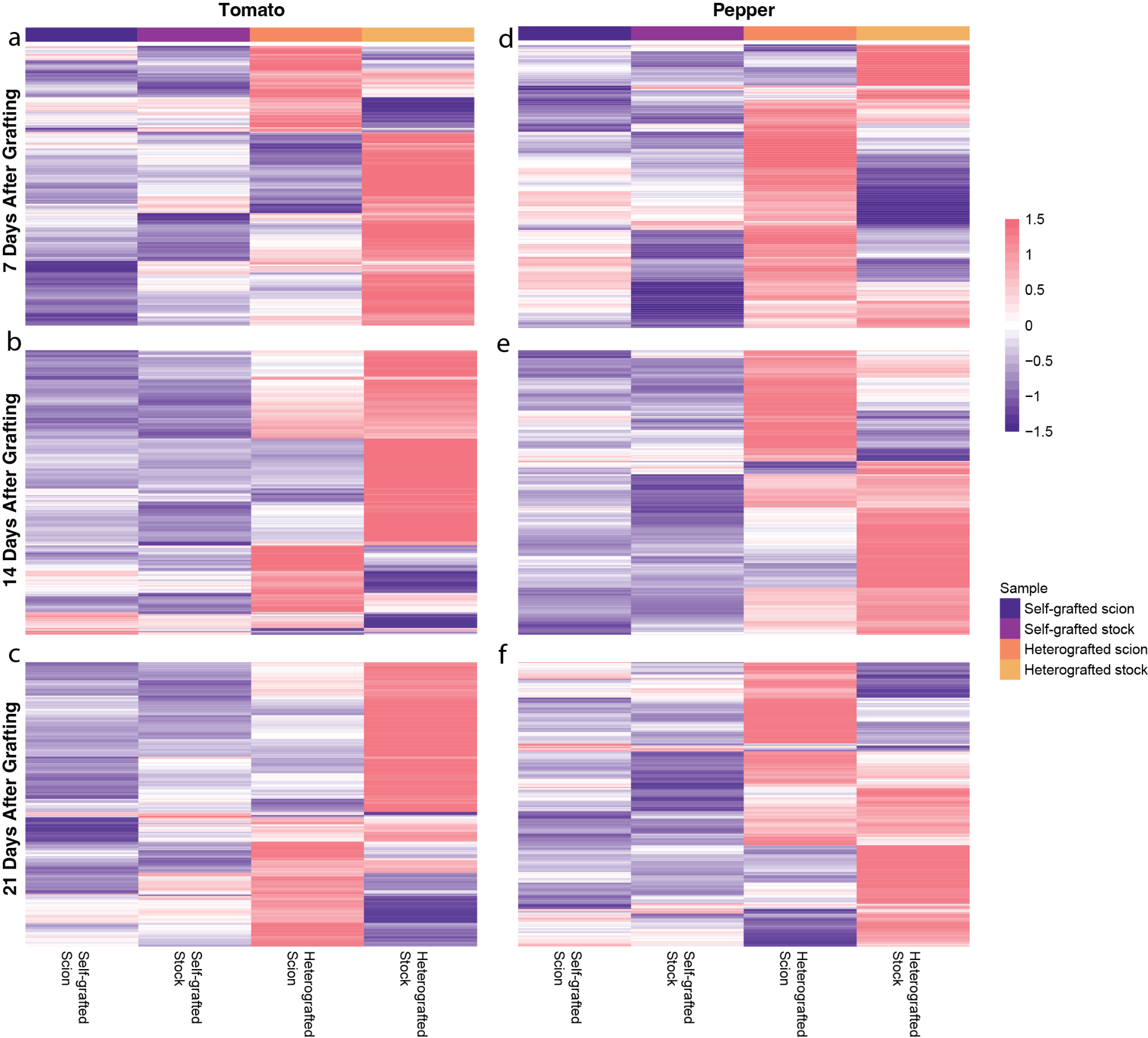


### Figure S12: Scion and stock tissue have distinct upregulated genes at any given time point

(a-c) Log fold change (LFC) of genes uniquely upregulated in tomato heterografts. (d-f) LFC of genes uniquely upregulated in pepper heterografts. Uniquely upregulated genes were determined by performing likelihood ratio testing (p<0.05) on ungrafted, self-graft scion, and heterografted scion tissue as well as ungrafted, self-grated stock, and heterografted stock tissue at each time point. The upregulated genes for each species-time category were combined and used to extract the log fold change of ungrafted versus grafted samples. The genes upregulated in only heterografted tomato tissue (scion or stock) at (a) 7, (b) 14, and (c) 21 DAG. The genes upregulated in only heterografted pepper tissue (scion or stock) at (d) 7, (e) 14, and (f) 21 DAG. The tissue is denoted by colored columns where self-grafted scions are dark purple, self-grafted stocks are light purple, heterografted scions are orange, and heterografted stocks are yellow. The LFC was scaled by row.


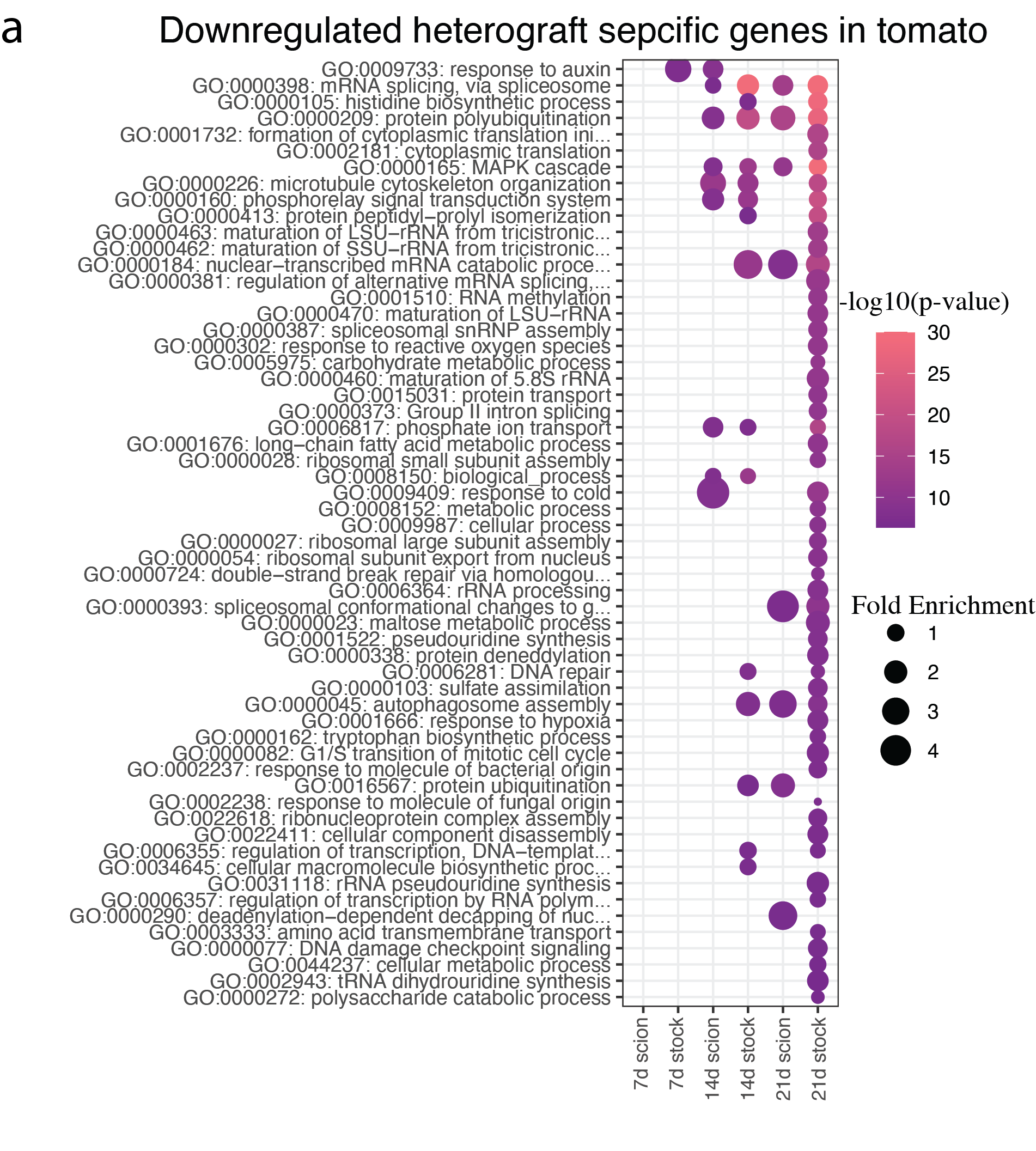


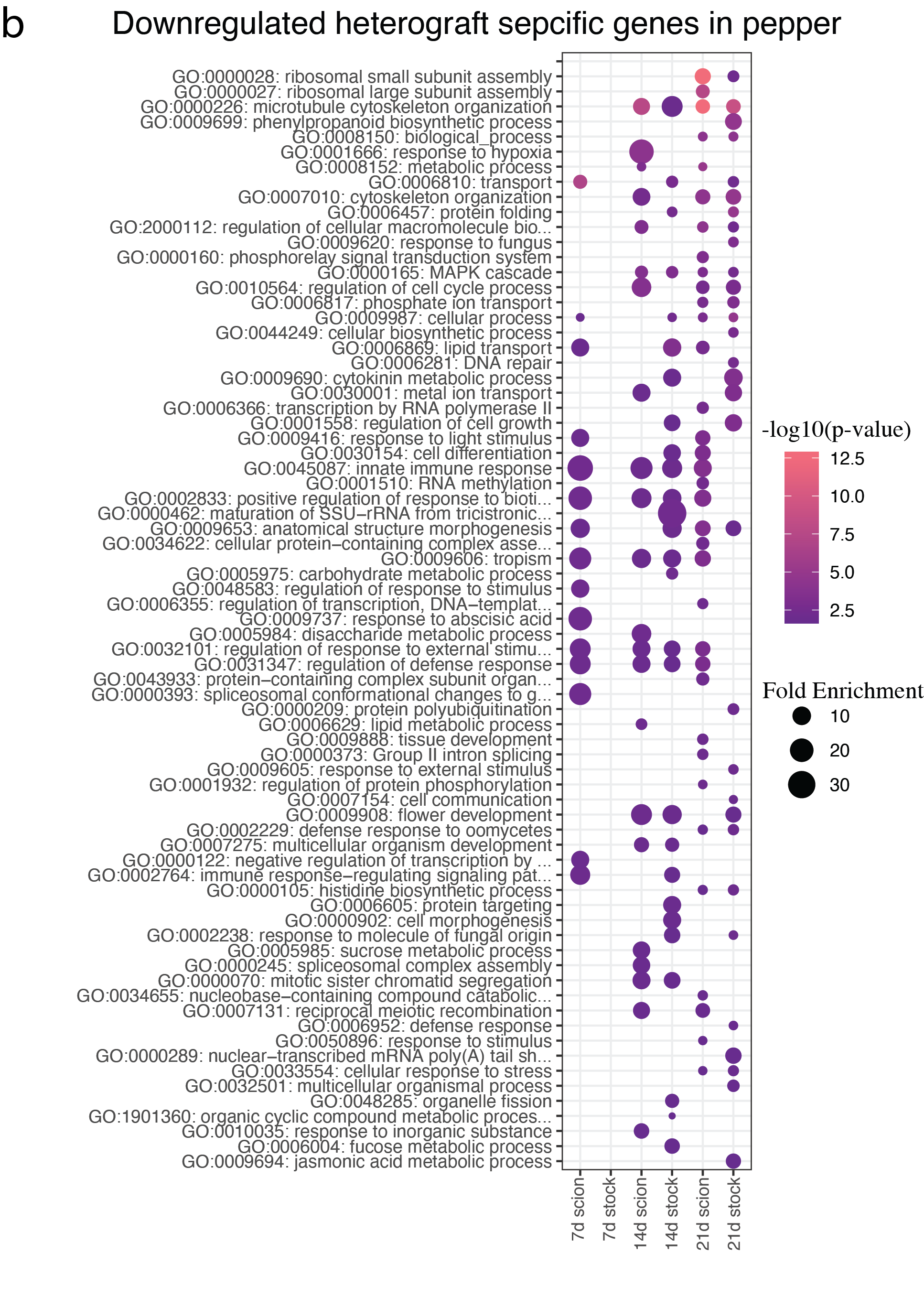


**Figure S13: Incompatible graft-specific downregulated genes GO enrichment**

**(**a-b) Uniquely downregulated incompatible graft genes were determined by performing likelihood ratio testing (p<0.05) on ungrafted, self-graft scion, and incompatible graft scion as well as ungrafted, self-grafted stock, and incompatible stock tissue. The genes downregulated in only the incompatible graft tissue were used to perform GO enrichment. GO terms enriched in downregulated incompatible grafted tomato tissue at 7, 14, and 21 DAG  (a). GO terms enriched in downregulated incompatible grafted pepper tissue at 7, 14, and 21 DAG (b).

**
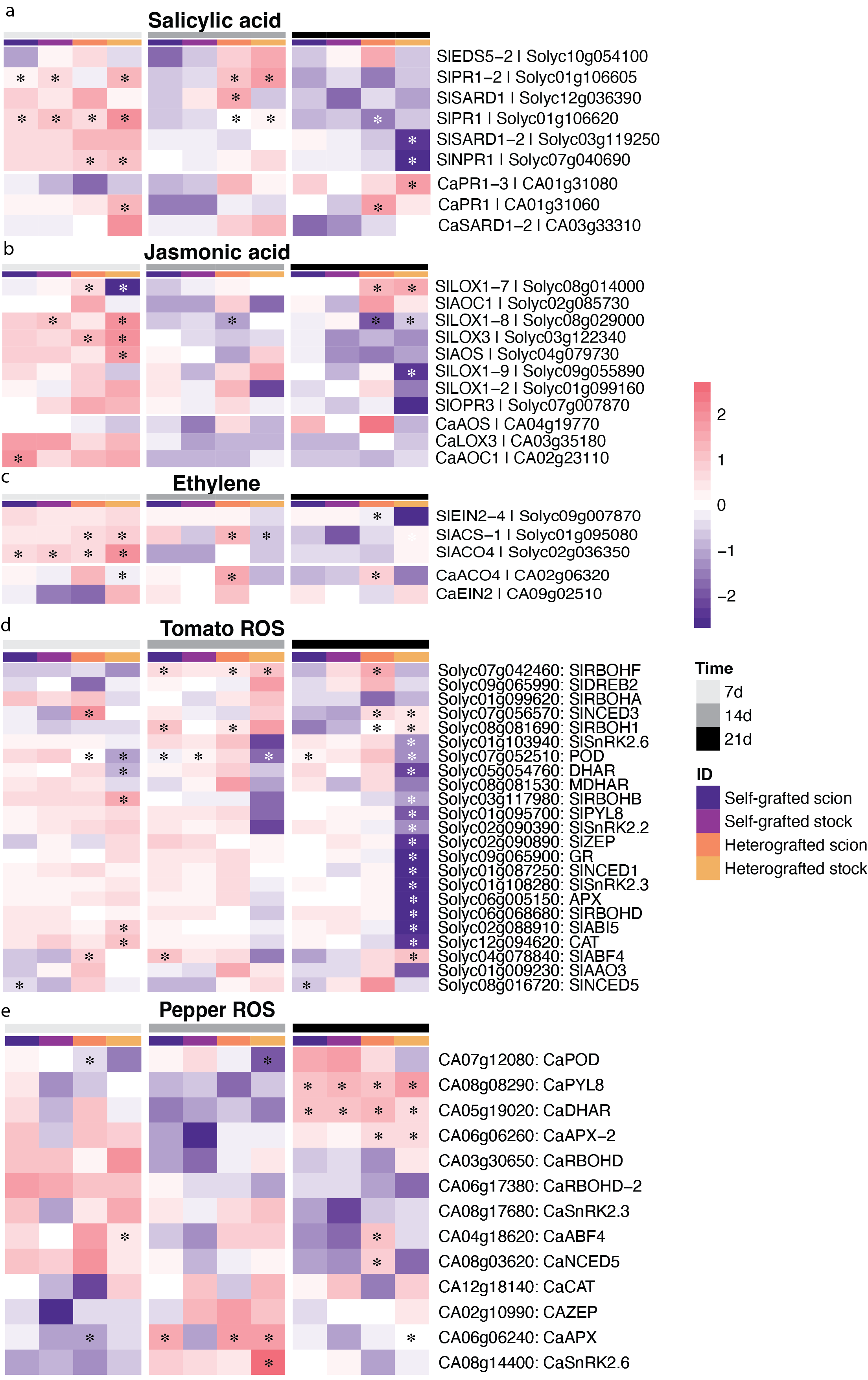
**

### Figure S14: Hormonal regulation but not ROS production is upregulated in incompatible grafts

(a-c) The LFC (ungrafted vs. grafted) of known genes involved in hormonal biosynthesis and signaling for salicylic acid (a), jasmonic acid (b), and ethylene (c). (d-e) The LFC (ungrafted vs. grafted) of genes involved in ROS and ABA signaling in tomato (d) and pepper (e). The tissue is denoted by colored columns where self-grafted scions are dark purple, self-grafted stocks are light purple, heterografted scions are orange, and heterografted stocks are yellow. The days after grafting are denoted by colored columns where 7 DAG are white, 14 DAG are grey, and 21 DAG are black. The LFC was scaled by row. LFC greater than |1.5| with an adjusted p-values less than 0.05 are notated by an asterisk.


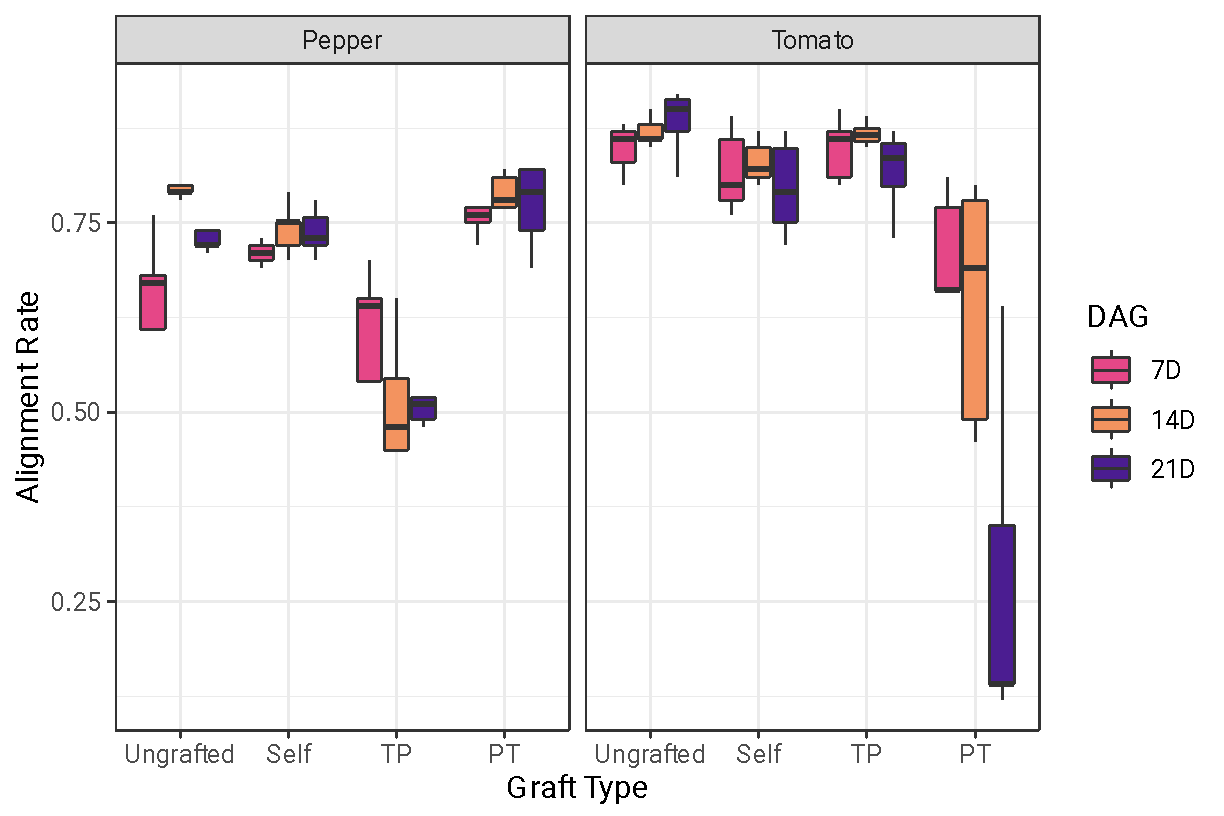


### Figure S15: RNA quality decreases over time in incompatible stocks

RNA quality can be correlated to alignment rates; Alignment rates were determined using STAR [(Dobin et al. 2013)](https://paperpile.com/c/mpVFWb/Kbiq). Transcripts from tomato were aligned to the *Solanum lycopersicum* (ITAG4.0) genome and transcripts from pepper were aligned to the *Capsicum annuum* (CM334) genome [(Kim et al. 2014; Hosmani et al. 2019)](https://paperpile.com/c/mpVFWb/EsX6+XldE2). Boxplots show the quadrant of data points, where pink is 7 DAG, orange is 14 DAG, and purple is 21 DAG.


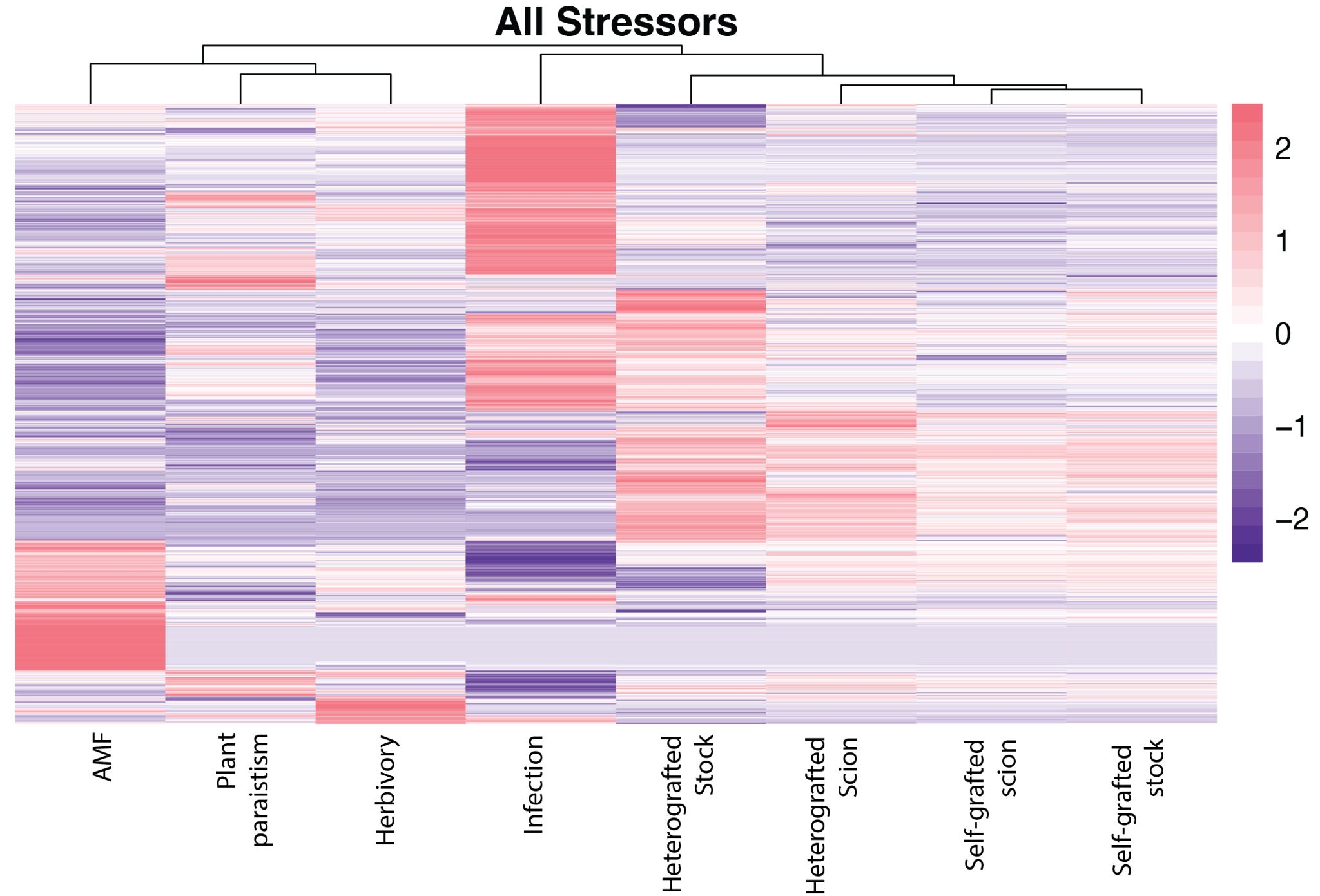


### Figure S16: Biological stressors upregulate distinct and shared genetic responses

Heatmap depicts the upregulated genes from the scion or stock of compatible grafts, scion or stock of incompatible grafts, symbiotic fungal colonization (AMF), plant parasitism, caterpillar herbivory, and necrotrophic fungi infection. All genes were pooled and the log-fold change between control and stressors were calculated and plotted. Gene expression was scaled by row and clustered using Complete distance.

### Methods S1: Plant material and growth conditions

To trigger germination, *Capsicum annuum, Capsicum chinense* (pepper), and *Solanum lycopersicum* (tomato) seeds were treated with 50% bleach for 30 seconds and then rinsed five times with sterile distilled water. Tomato seeds were germinated on wet paper towels in Phytotrays (Sigma-Aldrich) that were placed in the dark for 72 hours, transferred to the light for 72 hours, and then transplanted into Lambert LM-111 soil. Pepper seeds were sandwiched between two wet paper towels in Phyotrays and placed onto heat mats under 16:8 day/night light cycles for 7 days then transplanted into Lambert LM-111 soil. Tomato and pepper seedlings were grown in climate-controlled chambers set to 23 C with 16:8 day/night light cycles under F54T5/841/HO fluorescent bulbs (500-800 µmol/m^2^/sec).

### Methods S2: Grafting

*Capsicum sp.* seeds were grown as described above. Seven days later *Solanum lycopersicum* (Var. M82) seeds were grown as described above. Twenty-one-day-old pepper seedlings and 14-day-old tomato seedlings, which have the same stem diameter (1mm), were joined with a slant or wedge graft on the internode between the cotyledons and first leaf [(Kubota et al. 2008)](https://paperpile.com/c/mpVFWb/shndk). Grafts were held together with 1.5 mm silicon-top grafting clips (Johnny’s Selected Seeds, Albion, ME, USA). Grafted plants were generously watered, covered with plastic domes, and placed in the dark for 3 days. On day 4, plants were returned to light (500-800 µmol/m^2^/sec).

### Methods S3: Pepper compatibility grafts

100 *Capsicum annuum* var. California Wonder (CW), 100 RC Cayenne (Cayenne), 80 Doux des Landes (DDL), and 100 *Capsicum chinense* var. Habanero seeds were grown as described above. Seven days later, 200 *Solanum lycopersicum* (Var. M82) seeds were grown as described above. Grafts were performed in each of the following combinations: tomato:tomato, CW:CW, habanero:habanero, cayenne:cayenne, DDL:DDL, tomato:CW, CW:tomato, tomato:habanero, habanero:tomato, tomato:cayenne, cayenne:tomato, tomato:DDL, DLL:tomato. Plastic domes were vented 7 DAG and removed 14 DAG. The survival of various pepper-tomato graft combinations was monitored for 30 days (3, 7, 14, 21, and 30 DAG). 30 days after grafting the diameter of the scion and stem directly above the stem was measured using a digital caliper. The difference between the two measurements was noted. A subset of the surviving grafts were utilized for bend tests and vascular connectivity microscopy.

### Methods S4: Propidium Iodide Staining

Graft junctions were collected 30 DAG fixed in ice-cold Formalin-Alcohol-Acetic Acid (FAA) under a vacuum for 2 hours. After fixing in FAA, and dehydrating and rehydrating tissue, the samples were stained with 20 µg/ml propidium iodide (Acros Organics, CAS:25535-16-4) for 1 hour and rinsed with phosphate buffered saline. Tissue was then dehydrated again in the dark, and gradually transferred into methyl salicylate clearing agent. Finally, the tissue was cleared in 100% methyl salicylate at 4 C for 2 weeks. Fully cleared graft junctions were imaged on a Zeiss LSM880 Confocal Microscope using an Argon Laser 514 nm beam.

### Methods S5: Bend Test

Graft junction integrity was tested using manual bending as described [(Thomas et al. 2022)](https://paperpile.com/c/mpVFWb/y9EFt). 12 self-grafted tomato, 18 self-grafted CW, 6 self-grafted DDL, 22 self-grafted Habanero, 19 self-grafted cayenne, 10 tomato:CW, 9 CW:tomato, 13 tomato:DDL, 6 DDL:tomato, 6 tomato:Habanero, 5 Habanero:tomato, 9 tomato:cayenne, and 9 cayenne:tomato junctions were tested. Stems that broke at the graft junction were marked as broken, stems that did not break or broke at a different point of the stem were considered not broken.

### Methods S6: Instron three-point bend test

200 *Capsicum annuum* var. California Wonder (CW) seeds and 200 *Solanum lycopersicum* Var. M82 seeds were grown and grafted as described previously. These plants were transported to the University of Delaware 30 DAG for 3-point bending testing on an Instron 5943 (Norwood, Massachusetts USA) equipped with a 100 N load cell (Instron 2530 Series static load cell, Norwood, Massachusetts USA). [(Ennos, Crook, and Grimshaw 1993; Goodman and Ennos 2001)](https://paperpile.com/c/mpVFWb/Sgneh+vXpTV). Quantification of the mechanics of the graft junction was measured on a 17.5 mm span length custom 3-point bend fixture using a segment of the stem ranging from 18.7-29.8 mm in length [(Hostetler et al. 2022)](https://paperpile.com/c/mpVFWb/5SiHV). Stems were aligned so that the anvil was directly above the graft junction. Ungrafted and self-grafted plants were preloaded to 0.2 N; tomato:pepper grafts were preloaded to 0.01N and pepper:tomato grafts to 0.001N before data collection. The preload was reduced in inter-species grafts to capture the range of force required to displace the junction. All tests were performed by constant rate displacement of the top anvil at a rate of 1 mm/min, until junction failure or until the force-displacement curve leveled out. Force-displacement data were captured with Bluehill 3 software (Instron, Norwood, Massachusetts USA). 9 ungrafted tomato, 10 ungrafted tomato, 13 self-grafted tomato, 9 self-grafted pepper, 12 tomato:pepper, and 3 pepper:tomato plants were tested. Bio-replicates were limited in pepper:tomato grafts due to low survival rates. Furthermore, a two-sided power analysis (G*Power), which incorporated the mean k value, and standard deviation, was utilized to calculate the appropriate sample size [(Faul et al. 2009)](https://paperpile.com/c/mpVFWb/barOQ+JmTlm). The power analysis showed that a sample size of only 7 would be adequate to perform a t-test. We were unable to reach this value for all graft combinations but feel confident in our analysis due to the low standard deviation.

The force-displacement curves (N/mm) represent the structural stiffness (K) of the junction [(Hostetler et al. 2022)](https://paperpile.com/c/mpVFWb/5SiHV). The moment of inertia was calculated from caliper measurements of the graft junction geometry (Equation 1).

$I = \frac{\pi}{4} (a_{o}^{3}\times b_{o})$ Equation 1

where $\pi$ = 3.1415, a_o_ is the minor axis perpendicular to bending, and b_o_ is the major axis parallel to bending.

The structural bending modulus was calculated using K, the fixture span length (L, 17.5mm), and the moment of inertia (I) (Equation 2) [(Hostetler et al. 2022; Al-Zube et al. 2018)](https://paperpile.com/c/mpVFWb/5SiHV+JwNdk).

$Bending Modulus =K \times\frac{L^{3}}{48I}$ Equation 2

### Method S7: DAMP Assay

100 Tomato and 100 pepper seeds were sterilized and germinated on sterile seed germination medium as previously described [(Van Eck, Keen, and Tjahjadi 2019)](https://paperpile.com/c/mpVFWb/klb6). Pepper seeds were started 2 days before tomato. 7 days after sowing pepper and 5 days after sowing tomato, plants were removed from the media, and the hypocotyls of 50 tomatoes and 50 peppers were carefully cut into 3 mm pieces. Hypocotyls were placed in a 5x5 grid on fresh preculture medium [(Van Eck, Keen, and Tjahjadi 2019)](https://paperpile.com/c/mpVFWb/klb6). The exact placement of the tissue was noted by marking the plates with a permanent marker. The plates were placed in the dark at 22 C for 7 days. The explants were moved off of plates. Half of the tomato explants (n=25) were moved back onto a plate where tomato was previously cultured, making sure the tissue sits exactly on the previous marker line. The remaining tomato explants (n=25) were placed on plates that previously cultured pepper plants. Half the pepper plants were placed on plates that previously cultured tomato (n=25) and half the pepper plants were placed on plates that previously cultured pepper (n=25). The plates were returned to the dark (22 C) for 7 more days. After 14 days of culture total, the plates were imaged and the area of callus growth was quantified using ImageJ [(Van Eck, Keen, and Tjahjadi 2019; Schindelin et al. 2012)](https://paperpile.com/c/mpVFWb/klb6+dPvO5).

### Methods S8: Trypan Blue staining

*Capsicum annuum* var. California Wonder (CW) and *Solanum lycopersicum* Var. M82 were grafted as previously described. The junctions were cut from the plant with at least 1 cm of space between the cut and the graft junction to prevent the mechanical processes of cutting out the junction from interfering with cell death within the graft interface. Junctions were transferred into fresh 1% Trypan Blue and stained as previously reported (Fernández-Bautista, 2016). Junctions were cut transversely immediately preceding imaging on a Leica M205 fluorescent dissecting microscope using an EL6000 Mercury Metal Halide light source.

Images of Trypan Blue stained plants were adjusted for white balance. ImageJ was used to quantify areas of deep cell death [(Schindelin et al. 2012)](https://paperpile.com/c/mpVFWb/dPvO5). Regions with many layers of Trypan Blue stained tissue image as black. The blue channel was extracted, set to a threshold of 60-70/255, and the area of the deep tissue cell death was calculated. The total area of the junction used was determined by measuring 1.25 mm above and below the graft site. The width of the graft differed from plant to plant. The area of the junction was calculated based on a color threshold. The area of non-viable tissue, the area of the graft junction, and the percent of the junction containing non-viable tissue were calculated.

### Methods S9: TUNEL Assay

*Capsicum annuum* var. California Wonder (CW) and *Solanum lycopersicum* Var. M82 were grafted as previously described Junctions from 7, 14, and 21 DAG and ungrafted tissue of the same age. On each time point, 8 self-grafted tomato, 12 self-grafted pepper, 12 tomato:pepper, and 12 pepper:tomato graft junctions were collected. Similar regions of the stem were collected from 4 ungrafted tomato and 4 ungrafted pepper plants. The tissue was fixed in ice-cold FAA under a vacuum for 1 hour, then transferred to fresh FAA overnight at 4C. The tissue was dehydrated and then placed in 1:1 100% ethanol:polyester wax (Steedman’s Wax) kept at 37 C overnight. The tissue was moved through a series of 5 fresh 100% wax changes over 2 days. Wax and tissue were oriented in molds and tissue cassettes were immediately pressed against the wax. Wax blocks were allowed to solidify overnight at room temperature. Blocks were sectioned on Leica RM 2135 rotary microtome at 10 μm thickness. Ribbons were oriented on Poly-L-Lysine (Electron Microscopy Sciences, CAS 63410-01) coated slides. Room temperature water was pipetted on the slides and allowed to sit for 10 minutes to allow for expansion. Water was then removed, and slides were incubated flat at 40C for 10 minutes to ensure proper adhesion and allowed to dry overnight at room temperature.

Slides were de-paraffinized in 100% ethanol. Tissue was processed using the Promega DeadEnd™ Fluorometric TUNEL System according to the manual’s instructions. Steps were optimized for this experiment including adjusting the Proteinase K incubation time to 15 minutes and not utilizing the cover slips. The final tissue was counterstained in 1 ug/ml PI in PBS, coated in Vecta-shield mounting solution, and sealed under a coverslip using clear nail polish. The sides were either immediately imaged or kept at 4C overnight. Slides were imaged on a Zeiss LSM880 Confocal Microscope using an Argon Laser 514 nm beam. Fluorescein-tagged nuclei were viewed at 520 nm (nucleus of apoptotic cells and auto-fluorescent) and false color cyan. Propidium iodide was viewed at 620 nm (all nuclei and cell walls) and false color magenta.

### Method S10: RNA-sequencing and bioinformatic processing

*Capsicum annuum* v*ar. California Wonder* (CW) and *Solanum lycopersicum* *Var. M82* were grafted as previously described. A 0.5 cm piece of stem from the scion and stock were collected from 5 biological replicates for each sample. Young or heterografted plants could be separated by hand, while older more established grafts were cut down the graft junction with a clean razor blade. Each piece of tissue was flash frozen and ground with a mortal and pestle. Total RNA was purified from Trizol-extracted samples using the Monarch Total RNA Miniprep Kit (NEB #T2010) as described in their protocol. On-column DNase I treatment was performed as described in the kit protocol. RNA was eluted into 40 ul of water. The concentration and quality of the RNA was determined by Nanodrop and Fragment Analyzer. Libraries were built by the Cornell Institute of Biotechnology, Biotechnology Resource Centre using the Lexogen QuantSeq FWD prep kit and Poly(A) RNA Selection Kit V1.5 and subjected to high-throughput sequencing with the Illumina NextSeq 500/550 High-output kit. Fasta files were quality checked using FastQC 0.12.1 [(“Website,” n.d.)](https://paperpile.com/c/mpVFWb/Y0LjU) and TruSeq3 adaptors trimmed with Trimmomatic v0.39 [(Bolger, Lohse, and Usadel 2014)](https://paperpile.com/c/mpVFWb/Yrrqk) with a head crop setting of 12, trailing setting of 3, minimum length of 52, and a sliding window of 4:20.The polyA tail was trimmed using Prinseq v0.20.4 [(Schmieder and Edwards 2011)](https://paperpile.com/c/mpVFWb/opQVz) with the left and right tail length (i.e. trim_trail_left) of 8 and the minimum length of 40. Genome indexes were built for tomato and pepper using the ITAG4 [(Hosmani et al. 2019)](https://paperpile.com/c/mpVFWb/XldE2) and CM334 [(Kim et al. 2014)](https://paperpile.com/c/mpVFWb/EsX6) models respectively. Reads were aligned using STAR 2.7.10b [(Dobin et al. 2013)](https://paperpile.com/c/mpVFWb/Kbiq) with two pass mode. Tomato and pepper reads aligned at an average rate of 78% and 69%, respectively. Files were sorted using Samtools 1.18 [(Danecek et al. 2021)](https://paperpile.com/c/mpVFWb/VNpaY) and assembled using Strintie version 2.2.1 [(Pertea et al. 2015)](https://paperpile.com/c/mpVFWb/fmMZB). Differential expression analysis was performed using Deseq2 [(Love, Huber, and Anders 2014)](https://paperpile.com/c/mpVFWb/7LjNE) and Tidyverse (Wickham *et al*., 2019). Genes containing less than 10 reads on average or 0 reads in 75% of the samples were filtered from the data. Principle component analysis was performed on normalized samples, and outliers with low read counts were removed from the subsequent analysis. Correlations were determined using ggcorrplot (Kassambara, 2019) as Spearman correlation. Differential analysis was performed separately for each time point. Data was subset using Tidyverse and dplyr (Wickham *et al*., 2019, Wickham *et al*., 2023). Wald Tests were performed between ungrafted stems and self-grafted scions, self-grafted stocks, heterografted scions, and heterografted stocks. Significant differential expression was defined as an adjusted p-value less than 0.05 and a log fold change greater than 1.5 or less than -1.5. Likelihood ratio testing was performed on all samples at each time point and position (i.e. 7 DAG-scion would include self-grafted scions, heterografted scions, and ungrafted stem). From this comparison, significant genes were extracted, plotted, and clustered using DEGreport and lasso2 (Pantano, 2023, Lokhorst et al., 2021), where time was set to the sample, and col to null. Genes that showed a pattern of specific upregulation in the heterografted tissue were notated. The Wald lfc between the ungrafted tissue and heterografted tissue was determined for those genes across all tissue types. The lfc was plotted using pheatmap where the rows are scaled and clustered. The gene ontology of these genes was determined using topGO (Rahnenfuhrer, 2023). Gene2GO terms were generated using Blast2Go [(Conesa and Götz 2008)](https://paperpile.com/c/mpVFWb/5UUe). The CM334 pepper and ITAG4 tomato peptide sequences were utilized with default settings[(Kim et al. 2014)](https://paperpile.com/c/mpVFWb/EsX6). GO enrichment was determined using a topGO significance cut-off from the Weight01 algorithm and Fisher’s Statistic, with a p-value cut-off of 0.05.

### Method S11: Orthogroup Parsing

Orthogroups were determined using OrthoFinder with Diamond as the sequence search program, with an MCL inflation parameter of 5 for tight clustering (Emms and Kelly 2019; Buchfink et al. 2014). The *Solanum lycopersicum* (ITAG4), *Capsicum annuum* (CM334), and *Arabidopsis thaliana* (TAIR11) peptides sequences were used [(Kim et al. 2014; Hosmani et al. 2019a; Cheng et al. 2017)](https://paperpile.com/c/mpVFWb/khYy+5oCV+kD1F). For loose clustering between tomato and Arabidopsis, the previously published orthogroups were utilized [(Thomas et al. 2022)](https://paperpile.com/c/mpVFWb/y9EFt). Genes uniquely upregulated in the incompatible grafts were determined via LRT as previously described. Each of these DEGs, in both tomato and pepper, were assigned an ortho-grouping. The overlap between the two lists of orthogroups was parsed using R for each tissue-time combination (R Core Team 2020, Wickham 2011).

### Method S12: Comparative Transcriptomics

Publicly available RNA-seq data was downloaded using SRA-tools (https://github.com/ncbi/sra-tools/wiki) and fastq-dump (<https://ncbi.github.io/sra-tools/fastq-dump.html>). The samples were processed similarly to the original data with a few exceptions. Samples from [(Ke et al. 2021)](https://paperpile.com/c/mpVFWb/bvAp) and [(Zeng et al. 2023)](https://paperpile.com/c/mpVFWb/kRol) were paired-end and thus scripts were altered to adjust for this where needed. Samples from [(Lionetti and Vicré 2023)](https://paperpile.com/c/mpVFWb/43qB) were exceptionally short reads due to the nature of the collection, and thus the minimum length allowed for by Trimmomatic was lowered to 25 bp [(Lionetti and Vicré 2023; Bolger, Lohse, and Usadel 2014)](https://paperpile.com/c/mpVFWb/43qB+Yrrqk). All samples were aligned using the ITAG4 *Solanum lycopersicum* genome with HiSat2 [(Lionetti and Vicré 2023; Bolger, Lohse, and Usadel 2014; Kim et al. 2019)](https://paperpile.com/c/mpVFWb/43qB+Yrrqk+k9Ik). Differential analysis was performed as described in DESeq2, where Wald tests were conducted between the control and stressed treatments. The significance thresholds were set at p<0.05 and lfc greater than 1.5 and less than -1.5.

### Method S13: Statistical analysis

All statistical computation and graphical visualization generation was performed in R v.4.1.2 (R Core Team, 2023). Statistical significance of survival and stem integrity were calculated using Fisher’s Exact Test. Compact letter display was generated using rCompanion (Mangiafico, 2020). All interval data was tested for normality and homoscedasticity using Wilks-Shapiro Test and Levene’s Test from the CAR package [(Fox and Weisberg 2018)](https://paperpile.com/c/mpVFWb/1zRXQ). Non-parametric data was tested using the Kruskal–Wallis one-way analysis of variance in place of ANOVA and the Wilcoxon signed-rank test in place of Tukey's Honest Significant Difference test. DAMP assay results were tested for significance using a Paired T-test. Plots were made in R using ggplot2 and dplyr (R Core Team, 2023; Wickham 2016; Wickham 2023).

### References

**Alexa A, Rahnenfuhrer J. 2023**. topGO: Enrichment Analysis for Gene Ontology. [doi:10.18129/B9.bioc.topGO](https://doi.org/10.18129/B9.bioc.topGO), R package version 2.54.0, <https://bioconductor.org/packages/topGO>.

[**Al-Zube, Loay, Wenhuan Sun, Daniel Robertson, and Douglas Cook. 2018**. “The Elastic Modulus for Maize Stems.” *Plant Methods* 14 (February): 11.](http://paperpile.com/b/mpVFWb/JwNdk)

[**Andrews, S. 2010**. FastQC: A Quality Control Tool for High Throughput Sequence Data http://www.bioinformatics.babraham.ac.uk/projects/fastqc/](about:blank)[.](http://paperpile.com/b/mpVFWb/Y0LjU)

[**Bolger, Anthony M., Marc Lohse, and Bjoern Usadel. 2014**. “Trimmomatic: A Flexible Trimmer for Illumina Sequence Data.” *Bioinformatics*  30 (15): 2114–20.](http://paperpile.com/b/mpVFWb/Yrrqk)

[**Cheng, Chia-Yi, Vivek Krishnakumar, Agnes P. Chan, Françoise Thibaud-Nissen, Seth Schobel, and Christopher D. Town. 2017**. “Araport11: A Complete Reannotation of the Arabidopsis Thaliana Reference Genome.” *The Plant Journal: For Cell and Molecular Biology* 89 (4): 789–804.](http://paperpile.com/b/mpVFWb/kD1F)

[**Conesa, Ana, and Stefan Götz. 2008**. “Blast2GO: A Comprehensive Suite for Functional Analysis in Plant Genomics.” *International Journal of Plant Genomics* 2008: 619832.](http://paperpile.com/b/mpVFWb/5UUe)

[**Danecek, Petr, James K. Bonfield, Jennifer Liddle, John Marshall, Valeriu Ohan, Martin O. Pollard, Andrew Whitwham, et al. 2021.** “Twelve Years of SAMtools and BCFtools.” *GigaScience* 10 (2). https://doi.org/](http://paperpile.com/b/mpVFWb/VNpaY)[10.1093/gigascience/giab008](http://dx.doi.org/10.1093/gigascience/giab008)[.](http://paperpile.com/b/mpVFWb/VNpaY)

[**Dobin, Alexander, Carrie A. Davis, Felix Schlesinger, Jorg Drenkow, Chris Zaleski, Sonali Jha, Philippe Batut, Mark Chaisson, and Thomas R. Gingeras. 2013.** “STAR: Ultrafast Universal RNA-Seq Aligner.” *Bioinformatics*  29 (1): 15–21.](http://paperpile.com/b/mpVFWb/Kbiq)

**Ennos, A. R., M. J. Crook, and C. Grimshaw. 1993.** “The Anchorage Mechanics of Maize,Zea Mays.” [*Journal of Experimental Botany*](http://paperpile.com/b/mpVFWb/Sgneh)

[**Faul, Franz, Edgar Erdfelder, Axel Buchner, and Albert-Georg Lang. 2009**. “Statistical Power Analyses Using G*Power 3.1: Tests for Correlation and Regression Analyses.” *Behavior Research Methods* 41 (4): 1149–60.](http://paperpile.com/b/mpVFWb/JmTlm)

[**Fox, John, and Sanford Weisberg. 2018.** *An R Companion to Applied Regression*. SAGE Publications.](http://paperpile.com/b/mpVFWb/1zRXQ)

[**Goodman, A. M., and A. R. Ennos. 2001.** “The Effects of Mechanical Stimulation on the Morphology and Mechanics of Maize Roots Grown in an Aerated Nutrient Solution.” *International Journal of Plant Sciences*. https://doi.org/](http://paperpile.com/b/mpVFWb/vXpTV)[10.1086/320780](http://dx.doi.org/10.1086/320780)[.](http://paperpile.com/b/mpVFWb/vXpTV)

[**Hosmani, Prashant S., Mirella Flores-Gonzalez, Henri van de Geest, Florian Maumus, Linda V. Bakker, Elio Schijlen, Jan van Haarst, et al. 2019.** “An Improved de Novo Assembly and Annotation of the Tomato Reference Genome Using Single-Molecule Sequencing, Hi-C Proximity Ligation and Optical Maps.” *bioRxiv*. https://doi.org/](http://paperpile.com/b/mpVFWb/XldE2)[10.1101/767764](http://dx.doi.org/10.1101/767764)[.](http://paperpile.com/b/mpVFWb/XldE2)

[**Hostetler, Ashley N., Lindsay Erndwein, Elahe Ganji, Jonathan W. Reneau, Megan L. Killian, and Erin E. Sparks. 2022**. “Maize Brace Root Mechanics Vary by Whorl, Genotype, and Reproductive Stage.” *Annals of Botany*, March. https://doi.org/](http://paperpile.com/b/mpVFWb/5SiHV)[10.1093/aob/mcac029](http://dx.doi.org/10.1093/aob/mcac029)[.](http://paperpile.com/b/mpVFWb/5SiHV)

**Kassambara, Alboukadel, and Maintainer Alboukadel Kassambara. 2019.** "Package ‘ggcorrplot’." R package version 0.1 3.3

[**Ke, Lanlan, Yangzi Wang, Martin Schäfer, Thomas Städler, Rensen Zeng, Jörg Fabian, Hannier Pulido, Consuelo M. De Moraes, Yuanyuan Song, and Shuqing Xu. 2021.** “Transcriptomic Profiling Reveals Shared Signalling Networks Between Flower Development and Herbivory-Induced Responses in Tomato.” *Frontiers in Plant Science* 12 (September): 722810.](http://paperpile.com/b/mpVFWb/bvAp)

[**Kim, Daehwan, Joseph M. Paggi, Chanhee Park, Christopher Bennett, and Steven L. Salzberg. 2019.** “Graph-Based Genome Alignment and Genotyping with HISAT2 and HISAT-Genotype.” *Nature Biotechnology* 37 (8): 907–15.](http://paperpile.com/b/mpVFWb/k9Ik)

[**Kim, Seungill, Minkyu Park, Seon-In Yeom, Yong-Min Kim, Je Min Lee, Hyun-Ah Lee, Eunyoung Seo, et al. 2014.** “Genome Sequence of the Hot Pepper Provides Insights into the Evolution of Pungency in Capsicum Species.” *Nature Genetics* 46 (3): 270–78.](http://paperpile.com/b/mpVFWb/EsX6)

**Kubota, Chieri, Michael A. McClure, Nancy Kokalis-Burelle, Michael G. Bausher, and Erin N. Rosskopf. 2008**. “Vegetable Grafting: History, Use, and Current Technology Status in North America.” [*HortScience*. https://doi.org/](http://paperpile.com/b/mpVFWb/shndk)[10.21273/hortsci.43.6.1664](http://dx.doi.org/10.21273/hortsci.43.6.1664)[.](http://paperpile.com/b/mpVFWb/shndk)

[**Lionetti, Vincenzo, and Maïté Vicré. 2023.** *Plant Cell Wall in Pathogenesis, Parasitism and Symbiosis, Volume II*. Frontiers Media SA.](http://paperpile.com/b/mpVFWb/43qB)

**Lokhorst, Justin, Bill Venables, Berwin Turlach, and Maintainer Berwin Turlach. 2021.** "Package ‘lasso2’."

[**Love, Michael I., Wolfgang Huber, and Simon Anders. 2014.** “Moderated Estimation of Fold Change and Dispersion for RNA-Seq Data with DESeq2.” *Genome Biology* 15 (12): 550.](http://paperpile.com/b/mpVFWb/7LjNE)

**Mangiafico, Salvatore. 2020**. "rcompanion: Functions to support extension education program evaluation." R package version 2.10.

**Pantano L. (2023).** DEGreport: Report of DEG analysis. R package version 1.38.5, <http://lpantano.github.io/DEGreport/>.

[**Pertea, Mihaela, Geo M. Pertea, Corina M. Antonescu, Tsung-Cheng Chang, Joshua T. Mendell, and Steven L. Salzberg. 2015.** “StringTie Enables Improved Reconstruction of a Transcriptome from RNA-Seq Reads.” *Nature Biotechnology* 33 (3): 290–95.](http://paperpile.com/b/mpVFWb/fmMZB)

**R Core Team. 2023.** R: a language and environment for statistical computing, v.4.3.1. Vienna, Austria: R Foundation for Statistical Computing. [WWW document] URL [http://www.r-project.org](http://www.r-project.org/) [accessed 31 January 2023].

[**Schindelin, Johannes, Ignacio Arganda-Carreras, Erwin Frise, Verena Kaynig, Mark Longair, Tobias Pietzsch, Stephan Preibisch, et al. 2012.** “Fiji: An Open-Source Platform for Biological-Image Analysis.” *Nature Methods* 9 (7): 676–82.](http://paperpile.com/b/mpVFWb/dPvO5)

[**Schmieder, Robert, and Robert Edwards. 2011**. “Quality Control and Preprocessing of Metagenomic Datasets.” *Bioinformatics*  27 (6): 863–64.](http://paperpile.com/b/mpVFWb/opQVz)

[**Thomas, Hannah, Lisa Van den Broeck, Ryan Spurney, Rosangela Sozzani, and Margaret Frank. 2022.** “Gene Regulatory Networks for Compatible versus Incompatible Grafts Identify a Role for SlWOX4 during Junction Formation.” *The Plant Cell* 34 (1): 535–56.](http://paperpile.com/b/mpVFWb/y9EFt)

[**Van Eck, Joyce, Patricia Keen, and Michelle Tjahjadi. 2019**. “Agrobacterium Tumefaciens-Mediated Transformation of Tomato.” *Methods in Molecular Biology*  1864: 225–34.](http://paperpile.com/b/mpVFWb/klb6)

**Wickham H, Averick M, Bryan J, Chang W, McGowan LD, François R, Grolemund G, Hayes A, Henry L, Hester J et al. 2019**. Welcome to the tidyverse. Journal of Open Source Software 4: 1686.

**Wickham H. 2016**. *ggplot2: Elegant Graphics for Data Analysis*. Springer-Verlag New York. ISBN 978-3-319-24277-4, https://ggplot2.tidyverse.org.

**Wickham H, François R, Henry L, Müller K, Vaughan D. 2023**. dplyr: A Grammar of Data Manipulation. R package version 1.1.4, https://github.com/tidyverse/dplyr, [https://dplyr.tidyverse.org](https://dplyr.tidyverse.org/).

[**Zeng, Zhen, Yang Liu, Xing-Yu Feng, Sai-Xi Li, Xing-Mei Jiang, Jian-Qun Chen, and Zhu-Qing Shao. 2023**. “The RNAome Landscape of Tomato during Arbuscular Mycorrhizal Symbiosis Reveals an Evolving RNA Layer Symbiotic Regulatory Network.” *Plant Communications* 4 (1): 100429.](http://paperpile.com/b/mpVFWb/kRol)
